# Aqueous spinning of robust, self-healable, and crack-resistant hydrogel microfibers enabled by hydrogen bond nanoconfinement

**DOI:** 10.1038/s41467-023-37036-4

**Published:** 2023-03-13

**Authors:** Yingkun Shi, Baohu Wu, Shengtong Sun, Peiyi Wu

**Affiliations:** 1 State Key Laboratory for Modification of Chemical Fibers and Polymer Materials, College of Chemistry and Chemical Engineering & Center for Advanced Low-dimension Materials, Donghua University, Shanghai, 201620 China; 2 Jülich Centre for Neutron Science (JCNS) at Heinz Maier-Leibnitz Zentrum (MLZ) Forschungszentrum Jülich, Garching, 85748 Germany

**Keywords:** Gels and hydrogels, Structural properties, Gels and hydrogels

## Abstract

Robust damage-tolerant hydrogel fibers with high strength, crack resistance, and self-healing properties are indispensable for their long-term uses in soft machines and robots as load-bearing and actuating elements. However, current hydrogel fibers with inherent homogeneous structure are generally vulnerable to defects and cracks and thus local mechanical failure readily occurs across fiber normal. Here, inspired by spider spinning, we introduce a facile, energy-efficient aqueous pultrusion spinning process to continuously produce stiff yet extensible hydrogel microfibers at ambient conditions. The resulting microfibers are not only crack-insensitive but also rapidly heal the cracks in 30 s by moisture, owing to their structural nanoconfinement with hydrogen bond clusters embedded in an ionically complexed hygroscopic matrix. Moreover, the nanoconfined structure is highly energy-dissipating, moisture-sensitive but stable in water, leading to excellent damping and supercontraction properties. This work creates opportunities for the sustainable spinning of robust hydrogel-based fibrous materials towards diverse intelligent applications.

## Abstract (web summary)

Hydrogels with homogenous structure are vulnerable to defects and cracks, and local mechanical failure occurs consequently. Here, the authors develop a spinning process to produce robust hydrogel microfibers with both crack insensitivity and self-healability.

## Introduction

Nature produces diverse robust water-involving fibrous materials such as muscle fibers, nerves, and silks towards a range of different functionalities from safety, capture, water collection, sensing, to adaptive actuation^1^. These natural fibers have inspired the fabrication of numerous biomimetic hydrogel fibers with tailorable intelligent functions based on either polypeptides or non-peptide synthetic polymers^2–6^. However, due to the water-plasticizing nature and lack of hierarchical structure design, the mechanical performance of synthetic hydrogel fibers is often limited, which suffer from low strength and robustness to tolerate various damages. Although the mechanical resistance to global blunt damages (like stretching, impact, and bending) can be solved by introducing strong crosslinks and/or reinforcing elements, the vulnerability of hydrogel fibers to crack-related local damages (like cutting and tearing) has been long-term ignored yet an even more severe issue to limit their longevity. This is mainly owing to their inherent homogenous structure that generates a very small process zone on the order of atomic bonds surrounding cracks, which makes hydrogel fibers rather brittle and easy to create new cleavage surfaces^7–9^. Consequently, even very tiny defects (like cavities and flaws) generated from an erratic spinning process may cause catastrophic mechanical failure across hydrogel fiber normal. All the above concerns pose an urgent demand for crack-resistant hydrogel fibers with improved mechanical robustness in service, and even more challenging but essential, with potential crack self-healability to restore their original mechanical properties upon damage. Nevertheless, despite great efforts that have been made to produce various hydrogel fibers by 3D printing^10^, pultrusion/draw spinning^11–19^, wet spinning^2,20,21^, microfluidic spinning^22,23^, reactive spinning^24^, and tube templating^25,26^, none of these previous hydrogel fibers are reported to synergize the properties of high mechanical strength, crack resistance, and self-healability. Additionally, the complexity and organic solvents involving in many spinning processes also largely contradict to the energy-efficient aqueous production of natural fibers with ultralow carbon footprint^27^.

**Fig. 1:**
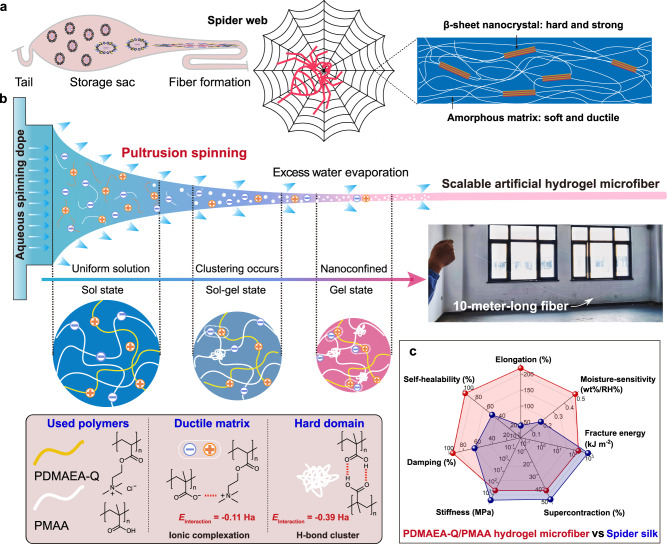
Schematic aqueous pultrusion spinning of robust hydrogel microfibers with hydrogen bond nanoconfinement. **a** Schematic spider spinning into high-performance silks with a hierarchical nanoconfinement structure that consists of highly conserved β-sheet nanocrystals as hard and strong domains as well as soft and ductile amorphous matrix. **b** Schematic continuous pultrusion spinning of PDMAEA-Q/PMAA hydrogel microfibers with spider silk-like nanoconfinement. With excess water evaporation at ambient conditions (25 °C, RH 60%), the gradual self-association of PMAA chains takes place generating high-density H-bond clusters embedded in the ionically complexed hygroscopic PDMAEA-Q/PMAA matrix. The calculated interaction energies (*E*_interaction_) of H-bond cluster and ionic complexation from molecular dynamics simulation are also shown (Ha = Hartree). Note that, the ion pair between PDAMEA-Q and PMAA is water-separated; however, for clarity, water molecules are not displayed in this paper. **c** A rough comparison of the stiffness, elongation, moisture sensitivity, self-healability, fracture energy, damping capacity, and supercontraction properties of the present hydrogel microfiber with spider silk (data in Supplementary Table 1). Source data are provided as a Source Data file.

Among natural fibers, spider silk represents almost the toughness limit of known biological materials with both high stiffness (3–10 GPa) and extensibility (15–45%)^28^. Ultralong silk fibers can be readily pulled out of the storage sac from highly concentrated aqueous spidroin dope in a fraction of a second. With the aid of hydrophilic proteins, spider silk also retains and reversibly absorbs water molecules leading to supercontraction for web recovery and enhanced vibrational signal transfer^29^. More importantly, in contrast to synthetic microfibers, spider dragline silk is reported to be highly crack-resistant to overcome the adverse effects of the existing defects which serve as seeds for material failure through localized stress concentration^30^. Theoretical and molecular dynamics simulation results ascribed the excellent crack tolerance of spider silk to the unique geometric nanoconfinement which comprises of 10–15 vol% hard β-sheet nanocrystals (size ≈2 nm; lattice spacing ≈10 nm) embedded in a soft semi-amorphous protein matrix (Fig. 1a). Such a hierarchical nanostructure not only achieves superhigh strength and toughness, but also enlarges the crack process zone size to 50 nm via the stress transfer through lattice deformation of confined nanocrystals, and thus decouples stress-loading events at different lengthy scales^7,31–34^. Moreover, the weak hydrogen bond (H-bond) nature and the very small size of β-sheet nanocrystals also allow spider silk to partially self-heal the ruptured flaws through the reformation of broken H-bonds in a stick-slip mode^33^. Such a mechanism has enlightened the biosyntheses of a few self-healing β-sheet-containing protein materials^35–37^. Reasonably, the incorporation of spider silk-like hydrogen bond nanoconfinement could be a feasible way to simultaneously impart the intriguing high mechanical strength, crack resistance, and self-healing properties to artificial hydrogel fibers.

In this paper, inspired by spider spinning and spider silk’s nanoconfined structure, we report the artificial synthesis of highly crack-resistant and self-healable robust hydrogel microfibers. Akin to spider spinning, scalable hydrogel microfibers can be continuously produced in an energy-efficient pultrusion spinning way at ambient conditions from an aqueous uniform dope that consists of poly(methylacrylic acid) (PMAA) and another hygroscopic, positively charged polyelectrolyte, poly(2-(dimethylamino)ethylacrylate) methyl chloride quarternary salt (PDMAEA-Q) (Fig. 1b). Upon excess water evaporation out of the spinneret, the spontaneous nanoconfinement (i.e. H-bond clustering) of PMAA chains naturally occurs. The formed strong H-bond clusters (assemblies of mainly dimeric H-bonds; interaction energy = −0.39 Ha) are finally embedded in a weak ductile water-retaining PDMAEA-Q/PMAA matrix (water-separated ion pair; interaction energy = −0.11 Ha) as separated nanophases, strongly resembling the nanoconfined two-phase structure of spider silk. Such a hierarchical nanoconfinement of the resulting hydrogel microfiber affords a high Young′s modulus of 428 MPa, elongation of 219%, and toughness of 19.8 MJ m^−3^ at ambient conditions (25 °C; relative humidity, RH, 60%). More intriguingly, a superhigh fracture energy of 187 kJ m^−2^ of the hydrogel microfiber that even surpasses woods, bones, and metal alloys is obtained which is directly related to the resistance to crack propagation. Furthermore, the highly dissipating physical network and moisture sensitivity bring ultra-rapid self-healing (in 30 s), highly damping (capacity ~95%), and supercontraction (43%) properties, which are unique to spider silk yet still difficult to realize in artificial microfibers (see rough comparison in Fig. 1c and Supplementary Table 1). Notably, although prepared from an aqueous spinning dope, the produced hydrogel microfibers do not dissolve in water again owing to the significant hydrophobic effect of the α-methyl groups of PMAA in stabilizing H-bond clusters, endowing them with excellent water stability towards potential applications in the diverse scenarios of soft machines and robots.

## Results

### Pultrusion spinning of hydrogel microfibers

**Fig. 2:**
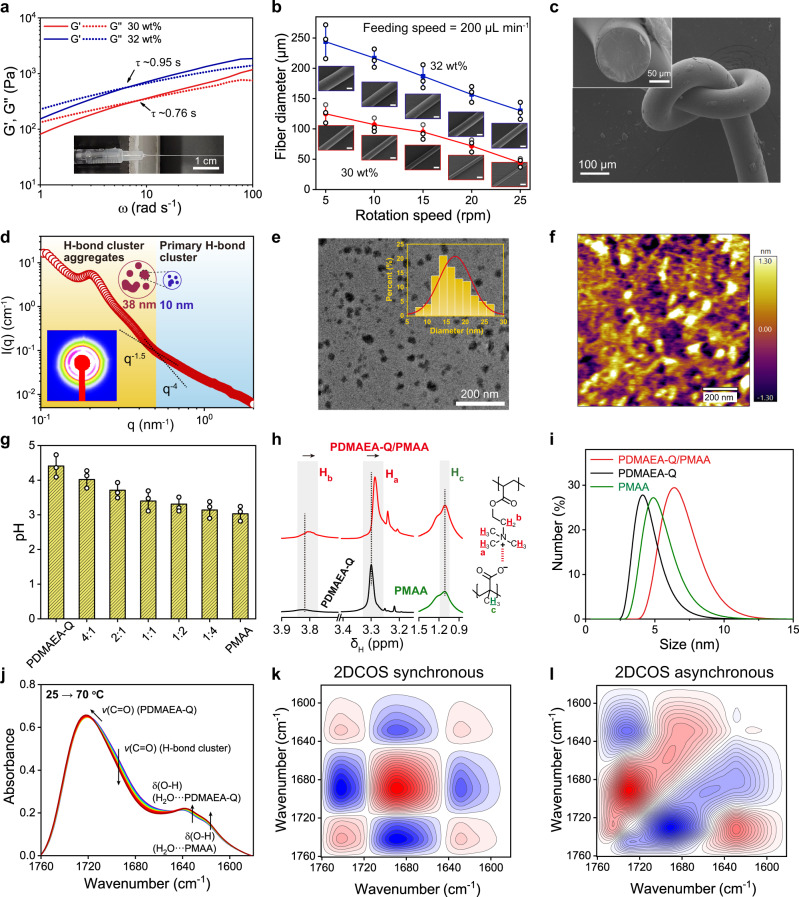
Morphology, nanoconfined structure, and internal interactions of PDMAEA-Q/PMAA hydrogel microfiber. **a** Rheological behavior of the spinning dopes with different polymer contents (τ: sol–gel relaxation time). **b** Fiber diameters as a function of rotation speeds and polymer contents in the dope (insets: corresponding SEM images; scale bar = 100 μm). **c**, SEM images of a knotted microfiber and the fractured cross-section. **d** SAXS curve and fitting results. **e**, **f** TEM and AFM height images show the presence of abundant H-bond clusters and cluster aggregates with the sizes of 8–28 nm. **g** pH values of PDMAEA-Q/PMAA mixtures with different DMAEA-Q:MAA molar ratios. **h** ^1^H NMR spectral comparison of PDMAEA-Q, PMAA, and their mixture (1:2 molar ratio) in D_2_O. For (g) and (h), the total monomer concentration was fixed to 0.1 M. **i** Corresponding DLS curves (concentration: 2.5 mM). **j** Temperature-dependent FTIR spectra and corresponding assignments of hydrated PDMAEA-Q/PMAA film (interval: 5 °C). **k**, **l** 2DCOS synchronous and asynchronous spectra generated from (**j**). The red colors represent positive intensities, while blue colors represent negative intensities. Fiber diameter and pH data are presented as mean values ± SD, *n* = 3 independent samples. Source data are provided as a Source Data file.

The spinning dope was prepared by the UV-induced polymerization of DMAEA-Q in the presence of high-molecular-weight PMAA (*M*_w_ ≈ 1 × 10^5^ g mol^−1^) (Supplementary Fig. 1). For pultrusion spinning, we delicately adjusted the rheological behavior of the spinning dope by tuning the molar ratios of DMAEA-Q and MAA as well as final polymer contents. It is found that the DMAEA-Q:MAA molar ratios no larger than 1:1 all allowed for the continuous spinning of ultralong hydrogel microfibers at ambient conditions. The 1:2 molar ratio was finally chosen as the optimal recipe due to the adequate glass transition temperature (*T*_g_ ≈ 63 °C, RH 60%) and high mechanical strength of the resulting fiber. Polymer content is another key factor to the rheology of spinning dope. As shown in Fig. 2a, the dope with 30 wt% polymer content responded as a highly viscous liquid over long timescales, and quickly became a solid-like gel over short timescales, suitable for pultrusion spinning^11^. The sol–gel relaxation time (*τ*) was 0.76 s derived from the crossover value of *ω* at which *G*′ = *G*″ (*ω* is the angular frequency, and *G*′ and *G*″ are the storage and loss moduli, respectively), approximate to that of spider’s major ampullate dope^38^. Slightly increasing polymer content to 32 wt% led to a little longer relaxation time (*τ* ~0.95 s), and as a result, thicker microfibers. Further increasing polymer content to 36 wt% or decreasing to 25 wt% would make the relaxation time either too long (>6 s) or too short (<0.2 s), both improper for continuous pultrusion spinning (Supplementary Fig. 2).

A rotating frame was used to collect the as-spun hydrogel microfibers which had a widely tunable diameter ranging from 30 to 250 μm depending on the dope rheology and rotation speed (Fig. 2b, Supplementary Fig. 3 and Movie 1; unless otherwise stated, the 125-μm-thick microfiber was used for all the following measurements). Ideally, the hydrogel microfiber could be spun to an infinite length if enough dope was supplied. Moreover, the hydrogel microfiber is highly transparent with ~98% transmittance in the visible spectral range (Supplementary Fig. 4). Scanning electron microscope (SEM) images of the surface and fractured cross-section show that the as-spun hydrogel microfiber is structurally compact yet flexible so as to be easily knotted (Fig. 2c). The uniform elemental distributions of O, C, and Cl atoms and the almost identical synchrotron infrared (IR) spectra at different positions further reveal the high quality of the resulting hydrogel microfibers (Supplementary Figs. 5 and 6).

It is highlighted that PMAA was carefully chosen instead of poly(acrylic acid) (PAA) owing to the presence of hydrophobic α-methyl groups that contribute to much stronger H-bond associations^39^. This is reminiscent of the structure of spidroins with alanine-rich domains forming highly conserved β-sheet nanocrystals while glycine-rich domains forming semi-amorphous matrix (alanine possesses an additional α-methyl group compared to glycine)^33^. The hydrophobic effect of α-methyl groups was verified by the higher initial water contact angle of PDMAEA-Q/PMAA film (105°) than PDMAEA-Q/PAA film (67°), in which PAA and PMAA have almost the same molecular weight (Supplementary Fig. 7). Immersed in water, PDMAEA-Q/PMAA film remained stable, but PDMAEA-Q/PAA film readily dissolved in 20 s (Supplementary Fig. 8), highlighting the significant role of α-methyl groups in stabilizing PMAA-related physical crosslinks. As a result, no continuous hydrogel microfibers could be successfully spun from the PDMAEA-Q/PAA dope. This may be ascribed to the weak H-bond crosslinking strength formed by PAA chains which cannot immediately stop the viscous flow out of the spinneret^11^. Accordingly, the cast PDMAEA-Q/PAA film is also mechanically weak with an ultimate tensile strength of only 45 kPa (Supplementary Fig. 9).

Meanwhile, a hygroscopic positively charged polymer like PDMAEA-Q is also necessary to accommodate spinnability for producing continuous hydrogel microfibers. Replacing DMAEA-Q by other positively charged monomers such as 3-(acrylamidopropyl)trimethyl ammonium chloride (APTMA) and 1-vinyl-3-ethylimidazolium bromide (ViEt) for the preparation of spinning dopes produced equally high-quality hydrogel fibers (Supplementary Fig. 10). However, using a negatively charged monomer, 3-sulfopropyl methacrylate potassium salt (SPMA), was not able to break the strong self-associated H-bonds of PMAA, resulting in stiff yet brittle fibers (Supplementary Fig. 11). Polymerizing zwitterionic monomers like 3-dimethyl(methacryloyloxyethyl) ammonium propane sulfonate (DMAPS) and 2-methacryloyloxyethyl phosphorylcholine (MPC) in the presence of PMAA led to gels or precipitates due to the strong association of polyzwitterions, and thus no spinnability was expected (Supplementary Fig. 12).

### Structural nanoconfinement and internal interactions

We performed small-angle X-ray scattering (SAXS) measurements to investigate the internal nanostructure of PDMAEA-Q/PMAA hydrogel microfiber. As shown in Fig. 2d, the 2D SAXS scattering pattern suggests a relatively weak orientation in the as-spun hydrogel microfiber. In the integrated 1D SAXS pattern, the power-law exponent is around −1.5 at high *q*, which reveals a primary molecular chain scattering that should originate from H-bond clusters. The *q*^−4^ scattering appears in the low *q* regime demonstrating the dense aggregation of H-bond clusters. The observed peak at around *q* = 0.2 nm^−1^ corresponds to an inter-distance of ~31 nm among H-bond clusters. SAXS curve fitting results indicate that the primary H-bond cluster has a mean size of 10 nm and cluster aggregates are larger than 38 nm. Transmission electron microscope (TEM) and atomic force microscope (AFM) observations strongly support the existence of high-density H-bond clusters with similar sizes as well as large cluster aggregates (Fig. 2e, f). AFM comparison of pure PDMAEA-Q and PMAA films suggests that H-bond clusters should only come from the self-association of PMAA chains while pure PDMAEA-Q is homogenous with very weak interchain associations (Supplementary Fig. 13). The absence of sharp diffraction peaks in the X-ray diffraction profile of PDMAEA-Q/PMAA reveals the amorphous nature of the microfiber, yet short-range order may exist owing to the presence of H-bond clusters (Supplementary Fig. 14).

The ionic complexation between PMAA and PDMAEA-Q is vital for the formation of ductile matrix surrounding hard H-bond clusters. To verify the presence of ionic complexation and evaluate the deprotonation degree of PMAA chains, we measured the pH values of PDMAEA-Q, PMAA, and their mixtures with different molar ratios. PDMAEA-Q is less acidic than PMAA, and their mixtures have the pH values just between them (Fig. 2g). Considering the pKa of PMAA is ~4.8, most of PMAA chains in the microfiber should be present in the protonated form (i.e. COOH), contributing to the formation of high-density H-bond clusters, while the remnant deprotonated PMAA groups (i.e. COO^−^) participate in the ionic complexation with the quaternary ammonium groups of PDMAEA-Q. Such an ionic complexation was supported by the ^1^H NMR spectral comparison among PDMAEA-Q, PMAA, and PDMAEA-Q/PMAA dissolved in D_2_O, in which all the complexation-related ^1^H resonances of PDMAEA-Q shifted to higher fields (Fig. 2h). The almost no chemical shift of the α-methyl groups of PMAA further revealed that most of PMAA chains are in the protonated state and not significantly affected by ionic complexation. Dynamic light scattering (DLS) of PDMAEA-Q/PMAA mixture showed an average chain size of 6.4 nm, larger than PDMAEA-Q (4.1 nm) and PMAA (4.8 nm) (Fig. 2i), suggesting again their association even in the solution state. Indeed, the SAXS results of the spinning dope had uncovered the ready presence of hierarchical nanofibrillar structures with the sizes between 6.5 and 30 nm (Supplementary Fig. 15). Reasonably, along with excess water evaporation in pultrusion spinning, the further growth of ionically complexed PDMAEA-Q/PMAA chains took place forming the final ductile matrix while the self-association of PMAA chains concomitantly occurred forming H-bond clusters.

To investigate the respective thermal sensitivities of different species in the hydrogel microfiber, we collected the temperature-dependent IR spectra of hydrated PDMAEA-Q/PMAA film (equilibrated at RH 60%) from 25 to 70 °C (Fig. 2j). Heating caused the slight dehydration of the ester C=O groups of PDMAEA-Q and the weakening of all the hydrogen bonds. The emergence of polymer-related water bending vibrations implies that the ion pairs between PMAA and PDMAEA-Q are water-separated with a weaker interaction strength than contact ion pairs^40^. 2D correlation spectroscopy (2DCOS) analysis was performed to elucidate the sequential order of these species upon heating. According to Noda’s judging rule based on the signs of cross-peaks at the synchronous and asynchronous spectra (Fig. 2k, l; see determination details in Supplementary Table 2)^41,42^, the responsive order of different groups to heat is as follows (→ means prior to or earlier than): 1630 cm^−1^ → 1615 cm^−1^ → 1691 cm^−1^ → 1730 cm^−1^, i.e. *δ*(OH) (H_2_O···PDMAEA-Q) → *δ*(OH) (H_2_O···PMAA) → *v*(COOH) (H-bond cluster of PMAA) → *v*(C = O) (PDMAEA-Q). This sequence suggests that the water molecules present in the PDMAEA-Q/PMAA ion pairs of amorphous matrix are thermally most labile, followed by H-bond clusters and hydrophobic ester groups. In other words, the water-separated ionic complexation is much weaker than the hydrogen bonds in H-bond clusters, and thus more easily affected by moisture. The almost constant wavenumber of *v*(COOH) of PMAA at RH 0% and 60% confirmed the moisture stability of H-bond clusters owing to the significant stabilizing effect of hydrophobic α-methyl groups (Supplementary Fig. 16).

The above strength sequence and moisture sensitivities of different interactions were supported by molecular dynamics simulation results. The interaction strength of four pairs, i.e. MAA···MAA, MAA···H_2_O···MAA, MAA···DMAEA-Q, and MAA···H_2_O···DMAEA-Q, was calculated (Supplementary Fig. 17). The interaction energy of MAA···MAA dimeric bond (−0.39 Ha) is much lower than that of water-separated MAA···H_2_O···DMAEA-Q ionic pair (−0.11 Ha), revealing the stronger association of H-bond cluster than ionic complexation in the microfiber. The moisture sensitivity of ionic complexation is evidenced by the significantly reduced absolute interaction energy of water-separated ionic pair (−0.11 Ha) compared to the direct contact pair (−0.57 Ha). In contrast, H-bond cluster is much stable against moisture with almost equal interaction energies with/without water (−0.36/−0.39 Ha).

### Tensile, damping and crack-resistant properties

**Fig. 3:**
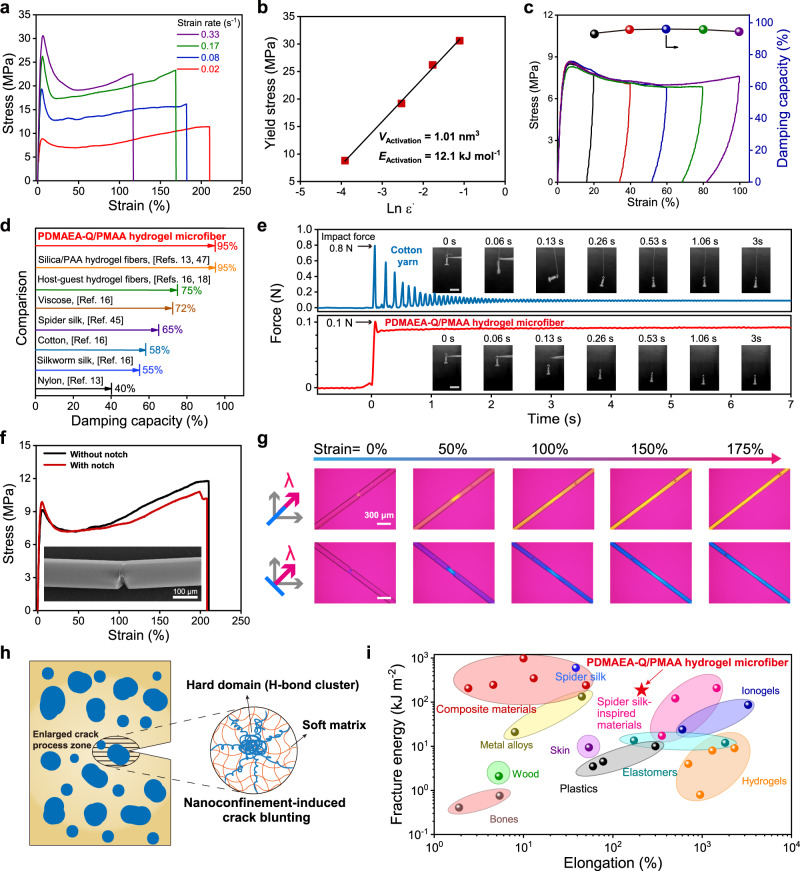
Tensile, damping, and crack-resistant properties of PDMAEA-Q/PMAA hydrogel microfiber. **a** Tensile behavior at different strain rates (RH 60%). **b** Linear fitting of yield stresses as a function of the natural logarithm of strain rates (ε̇). **c** Cyclic loading-unloading curves and corresponding damping capacities with increasing strains (strain rate = 0.02 s^−1^). **d** Comparison of the damping capacity of the hydrogel microfiber with other typical damping fiber materials. **e** Time-resolved impact force oscillations of a free-falling object buffered by cotton yarn and the hydrogel microfiber, respectively. **f** Tensile curves of the microfiber with/without a notch (inset: SEM image of the notched microfiber). **g** POM images of a notched microfiber as stretched from 0% to 175% strains. The images were taken in the presence of a 530 nm tint plate at the azimuth angles of 45° and −45^o^, respectively. **h** Proposed mechanism for crack tolerance. **i** Comparison of the fracture energies and elongations among the hydrogel microfiber and other materials (data in Supplementary Table 3). Source data are provided as a Source Data file.

Similar to many natural fibers, PDMAEA-Q/PMAA hydrogel microfiber is extensible with very high mechanical strength. At the strain rate of 0.02 s^−1^, the hydrogel microfiber equilibrated at RH 60% exhibits the Young′s modulus, elongation, tensile strength, and toughness to be 428 MPa, 219%, 11.6 MPa, and 19.8 MJ m^−3^, respectively (Fig. 3a; see the fiber’s load-bearing capacity in Supplementary Movie 2). The tensile behavior of hydrogel microfiber was highly reproducible, demonstrating the high spinning quality (Supplementary Fig. 18). An apparent yield point at about 5% strain was observed, suggesting that the hydrogel microfiber is in a glassy state at ambient conditions. Increasing strain rates led to the reduction of maximum strains as well as higher tensile strengths and yield stresses. The relationship between yield stress and the natural logarithm of strain rate is close to linear (Fig. 3b), in line with the Eyring model of force-induced dissociation of noncovalent bonds^43^. The fitted activation volume was 1.01 nm^3^, which can be comprehended as the segmental size of the amorphous matrix involved in the motion associated with yielding. Besides, the apparent activation energy was calculated to be 12.1 kJ mol^−1^, which is assessed to be the energy barrier to overcome the mobile segments. After the yield point, the long plateau of plastic deformation is mainly ascribed to the slide-on extension of amorphous matrix as confined by H-bond clusters. At the strains larger than 100%, strain stiffening occurred which can be attributed to the gradual unfolding of H-bond clusters until complete failure, analogous to the cases in spider silk and H-bond-involving hydrogels^42,44^. Further increasing the PMAA ratios in the hydrogel microfiber led to higher Young′s moduli and reduced elongations, and vice versa (Supplementary Fig. 19).

The hidden large energy dissipation in the long plastic deformation brings excellent damping capacity to the hydrogel microfiber. Damping describes how oscillations in a system decay after a disturbance. For example, spider silk scarcely oscillates upon impact owing to the dissipating extension through chain sliding, corresponding to a relatively high damping capacity of 50–70% (measured by the ratio of dissipated energy to stored energy)^45,46^. Intriguingly, the hydrogel microfiber exhibits an extremely high damping capacity of ~95% with apparent hysteresis loops in the strain range of 20–100% (Fig. 3c). Upon stretching to 100% strain, the dissipated energy can reach as high as 7 MJ m^−3^. Such a high damping capacity has far exceeded that of spider silk as well as most of natural and synthetic fibers (see comparison in Fig. 3d)^13,16,18,45,47^. To demonstrate the potential damping application in shock-absorbing scenarios, we monitored the time-resolved force oscillations induced by a free-falling object (10 g) that was tied to a 4-cm-long hydrogel microfiber and dropped from a height of 8 cm. At the same conditions, a cotton yarn (length: 8 cm; diameter: 500 μm) with a typical damping capacity of ~58% generated a maximum impact force of 0.8 N, 7 times higher than the hydrogel microfiber (0.1 N) (Fig. 3e). Moreover, the decay of the oscillating force for the hydrogel microfiber was also much faster, on which the tied object rapidly calmed down after free falling.

We further demonstrate that the hydrogel microfiber is highly crack-resistant like spider silk. According to pure shear test^48^, a 30-μm-deep single-edge cut was created by a sharp blade in the middle of the microfiber, and tensile test was then performed to calculate the critical strain at which crack started to propagate. The critical strain of the notched microfiber is approximate to the maximum elongation of the unnotched one (Fig. 3f), suggesting that the hydrogel microfiber is almost insensitive to cracks. To visualize crack propagation, we employed polarized optical microscope (POM) to observe the strain-induced stress distribution of the notched microfiber via interference color changes. As stretched, the polarized images at the azimuth angles of 45° and −45° showed orange and blue colors, respectively, which suggests the strong orientation of the microfiber along the stretching direction (Fig. 3g)^42^. Apparent stress concentration at the crack tip could be observed at 50% strain, which however was gradually eliminated in the following strains. We ascribed the excellent crack resistance of the hydrogel microfiber to the spider silk-like nanoconfined hierarchical structure, in which cracks are quickly pinned and blunted by high-energy hard domains via the stress transfer and reorientation of H-bond clusters (Fig. 3h)^31,49^. The nanoconfined structure is expected to enlarge the crack process zone from the atomic subnanometer scales (<1 nm) for a homogeneous structure to much larger nanoscales (>30 nm) through the lattice deformation of interconnected H-bond clusters^7^. The fracture energy of the hydrogel microfiber for quantitatively evaluating crack resistance was calculated to be 187 kJ m^−2^, which has surpassed many kinds of soft and hard materials including bones, woods, and metals/alloys, and also ranked in the top level of spider silk-inspired crack-resistant materials (see comparison in Fig. 3i and Supplementary Table 3; data for dragline spider silk are presented in Supplementary Fig. 20).

### Moisture sensitivity and self-healability

**Fig. 4:**
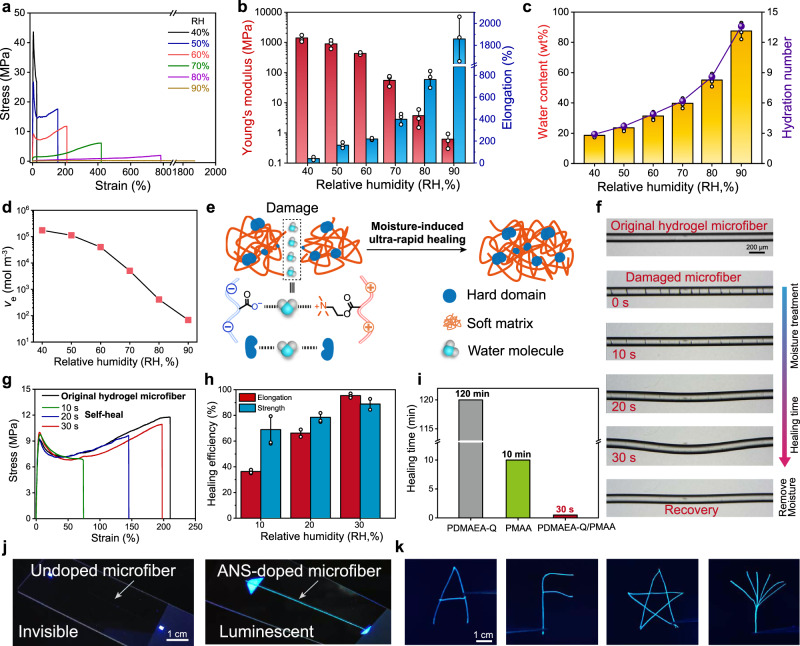
Moisture sensitivity and self-healability of PDMAEA-Q/PMAA hydrogel microfiber. **a** Tensile stress-strain curves at different humidities. **b** Corresponding Young′s moduli and elongations. **c**, **d** Humidity-dependent equilibrium water contents, hydration numbers, and effective crosslinking densities. **e** Schematic mechanism for moisture-induced ultra-rapid self-healing of the hydrogel microfiber which relies on the chain diffusion and reformation of both ionic complexations and H-bond clusters. **f** Optical images of moisture-induced rapid self-healing of multiple cracks on the microfiber. **g** Tensile curves of self-healed hydrogel microfibers at different healing time. **h** Time-dependent healing efficiencies of elongation and tensile strength. **i** Comparison of the healing time among PDMAEA-Q/PMAA, PDMAEA-Q, and PMAA microfibers. **j** Photos of original and ANS-doped hydrogel microfibers under UV irradiation. **k** Fluorescent images of fused hydrogel microfibers with different architectures. Young′s modulus, elongation, water content, and healing efficiency data are presented as mean values ± SD, *n* = 3 independent samples. Source data are provided as a Source Data file.

Owing to the presence of hygroscopic PDMAEA-Q, the mechanical properties of PDMAEA-Q/PMAA hydrogel microfiber can be substantially altered by environmental humidity. Reducing RH to below 40% caused the microfiber to be rather stiff but fragile, while increasing RH led to a more extensible microfiber with decreased moduli and higher elongations (Fig. 4a, b). When RH is above 80%, the yield point totally vanished, corresponding to a highly elastic microfiber. At RH 80%, the J-shaped true stress–strain curve characteristic for strain stiffening indicates that even at such a high humidity, the H-bond clusters remained stable serving as strong crosslinks to restrain mechanical failure at large strains (Supplementary Fig. 21)^42^. Further increasing RH to 90% led to the diminishing of strain stiffening behavior yet a very large elongation of 1858%, suggesting that the H-bond clusters in the hydrogel microfiber can still be water-plasticized at enough high humidities. Even so, similar to PDMAEA-Q/PMAA film, immersing the hydrogel microfiber in water for 24 h did not induce any dissolving event, highlighting again the important role of hydrophobic α-methyl groups in stabilizing PMAA-related physical crosslinks (Supplementary Fig. 22). To recover the sol state for re-spinning, the addition of HCl as counterions was needed to screen the ionic complexation between PDMAEA-Q and PMAA.

The moisture sensitivity of hydrogel microfiber is closely related to the equilibrium water content and hydration numbers (defined as the number of water molecules per carboxylic acid group). By increasing RH from 40% to 90%, the water content increased drastically from 18.6 wt% to 87.5 wt%, and corresponding hydration number from 2.9 to 13.6 (Fig. 4c). Compared to spider silk, PDMAEA-Q/PMAA hydrogel microfiber has both much higher water contents and moisture sensitivities in the whole humidity range (Supplementary Fig. 23). Moreover, the high water content and mobile ions (H^+^ and Cl^-^) also provided hydrogel microfiber with good ionic conductivity (3.9 mS m^−1^ at RH 60%; Supplementary Fig. 24). It is expected that the absorbed water molecules would greatly reduce the physical crosslinking strength of the amorphous matrix, and thus plasticize the whole microfiber. We calculated the effective crosslinking densities of the hydrogel microfiber at different humidities according to classical rubber elasticity theory^50^. The effective crosslinking density decreased remarkably from 17.3 kmol m^−3^ (RH 40%) to 69 mol m^−3^ (RH 90%) (Fig. 4d), which strongly consolidates the above conclusion.

The unique moisture sensitivity allows the hydrogel microfiber to self-heal ultra-rapidly, because both the ionic complexation in the amorphous matrix and hydrogen bonds in the H-bond clusters are readily regenerated with the aid of water molecules (Fig. 4e). To evaluate the self-healing capacity, we monitored the changes of multiple cracks on the microfiber as exposed to a large amount of moisture produced by a humidifier (Fig. 4f and Supplementary Movie 3). All the cracks on the microfiber quickly disappeared in 30 s, suggesting its excellent self-healability. Note that, the hydrogel microfiber became elongated by further absorbing water in the healing process, yet fully recovered as exposed to the environmental low humidity again. This reveals that the physically crosslinked network was not significantly disrupted during healing, and thus the mechanical integrity could be well retained, which takes a major advantage over other healing chemistries based on solid-to-liquid transitions that cause the remarkable loss of mechanical strength during healing^51^.

Moreover, the moisture-induced self-healing process healed not only the damaged morphology but also mechanical properties in an ultra-rapid way (Fig. 4g). Healing for 30 s had already restored 96% elongation and 89% tensile strength of the hydrogel microfiber (Fig. 4h). It is highlighted that such a rapid self-healing feature is unique to the hydrogel microfiber, while the apparent crack healing of pure PDMAEA-Q and PMAA fibers needed much longer time (120 and 10 min, respectively) (Fig. 4i and Supplementary Fig. 25). This is believed to originate from the stabilized nanoconfined structure of the hydrogel microfiber, since the global network structure does not need to reform and only the interfacial reconfiguration through chain diffusion within amorphous matrix and H-bond clusters is required which facilitates fast healing^35^. In contrast, in pure PMAA fiber, the intermolecular hydrogen bonds are too strong to allow polymer chains to rapidly diffuse, while for pure PDMAEA-Q fiber, the probable chemical cross-linking through chain transfer reaction in the polymerization hindered its fast self-healing (the fiber only swelled in water).

The moisture-induced self-healing advantage makes the fusion of different hydrogel microfibers possible, which is promising in deriving high-level assembled fibrous substructures^1^. To make the hydrogel microfiber visible in the dark, we functionalized the microfiber by doping a fluorescent agent, 8-anilinonaphthalene-1-sulfonic acid (ANS), into the spinning dope, which is known to exhibit strong fluorescence in the hydrophobic environment^52^. Owing to the presence of hydrophobic α-methyl groups, ANS-doped hydrogel microfiber emitted strong blue fluorescence under the irradiation of 365 nm UV light (Fig. 4j). Assembling the fluorescent microfibers in different architectures and then treating them with moisture allowed the joints to rapidly fuse together, resulting in free-standing hydrogel fiber substructures that can be clearly observed in the dark (Fig. 4k).

### Supercontraction and shape-memory

**Fig. 5:**
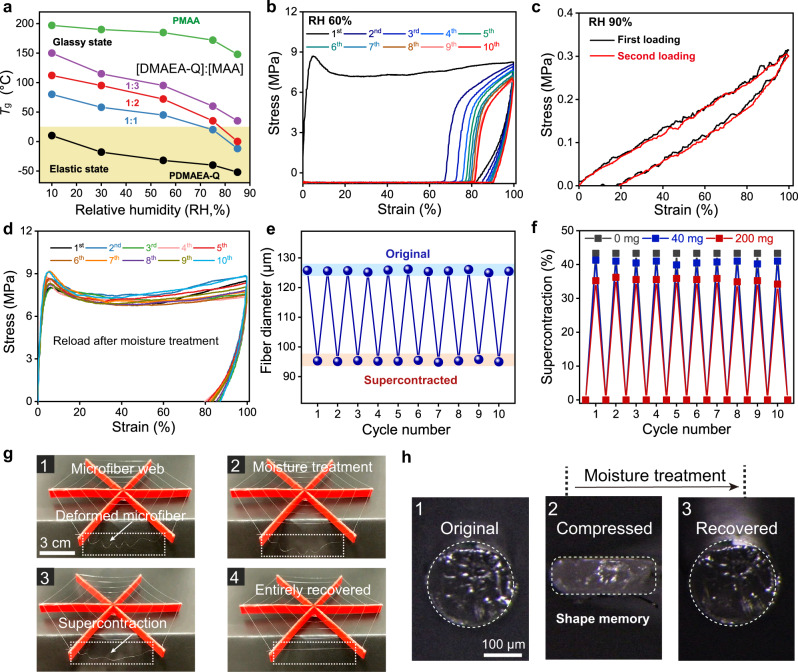
Supercontraction and shape-memory properties of PDMAEA-Q/PMAA hydrogel microfiber. **a** Glass transition temperatures (*T*_g_) with varying DMAEA-Q:MAA molar ratios and humidities. **b** Tensile loading-unloading curves at RH 60% for 10 cycles (waiting time = 0 s). **c** Loading-unloading curves at RH 90% for two cycles (waiting time = 30 s). **d** Loading-unloading curves at RH 60% for 10 cycles of moisture treatments. **e** Repeatable fiber diameter changes in the moisture-induced supercontraction process for 10 cycles. **f** Load-dependent supercontraction ratios. **g** Demonstration of the supercontraction effect by a hydrogel microfiber web. **h** Shape-memory behavior of the hydrogel microfiber in the compressive mode. Source data are provided as a Source Data file.

Humidity-induced supercontraction is a well-known property of spider silk but rarely observed in other materials^16^. After being deformed by a prey or other objects, the unrestrained spider web can recover its original shape in the rain or dew, owing to the entropy-driven recoiling of amorphous matrix which turns the silk into a rubber^53^. Here we show that the glassy and elastic states of PDMAEA-Q/PMAA hydrogel microfiber can also be reversibly converted in response to humidity. Increasing either molar ratio or humidity caused the reduction of the glass transition temperatures of hydrogel microfibers (Fig. 5a and Supplementary Fig. 26). Using the room temperature of 25 °C as the reference, only the hydrogel microfibers with the DMAEA-Q:MAA molar ratios of 1:2 and 1:1 can be switched between the glassy and elastic states in the RH range of 10–90%. For the studied microfiber with 1:2 molar ratio, the critical humidity is ca. RH 78%. That means, below and above RH 78%, stretching the hydrogel microfiber caused irreversible plastic and reversible elastic deformations, respectively. Such a humidity-induced switching of mechanical properties was confirmed by the cyclic tensile curves at RH 60% and 90%, respectively (Fig. 5b, c).

At RH 60%, the residual strain is about 80% after stretching to 100% strain, which can be repeated for many times after consecutive humidifying-drying treatments (Fig. 5d and Supplementary Fig. 27). The coincidental curves with almost the same hysteresis loops suggest that moisture can reset the fiber’s properties, which was also observed in other supercontractive fibers^16^. To be specific, the hydrogel microfiber was first stretched to a fixed length and after removing the restrain, the fiber would automatically contract by 20%; then, moisture treatment can fully recover the residual 80% strain by turning the fiber into an elastomer. The supercontraction-related diameter changes of the hydrogel microfiber were fully reversible (Fig. 5e and Supplementary Fig. 28). The hidden supercontraction mechanism of the hydrogel microfiber is similar to spider silk, i.e. the entropy-driven recoiling of the amorphous phase takes place in response to humidity while H-bond clusters act as strong crosslinks to ensure full elastic recovery. Such a mechanism was supported by the POM images of hydrogel microfiber in the whole supercontraction process, in which stretch-induced structural orientation can be fully eliminated by moisture treatment (Supplementary Fig. 29).

The supercontraction ratios of the hydrogel microfiber are dependent on the load, which in turn could act as an artificial muscle for weight lifting (Supplementary Fig. 30 and Movie 4). Without load, the supercontraction ratio was estimated to be 43% (Fig. 5f), comparable to that of spider silk (~50%)^6^. Increasing the load to 40 and 200 mg would decrease the supercontraction ratio to 41% and 35%, respectively, due to the reduced recoiling degree in the presence of biased stress. The energy density of work done by hydrogel microfiber was calculated to be 96 J kg^−1^, much higher than that of mammalian skeletal muscle (39 J kg^−1^)^2^. For demonstration, we winded several hydrogel microfibers on a supporting plastic frame like a spider web, and a length of the microfiber was first stretched and then released which spontaneously coiled due to irreversible plastic deformation (Fig. 5g and Supplementary Movie 5). Moisture treatment induced the rapid supercontraction of the fiber and thus recovered the whole web, resembling real spider web^29^.

It is noted that supercontraction is actually a shape-memory behavior in the tensile shape-recovering mode. We found that the hydrogel microfiber was also shape-memorized in the compressive mode. Compressing the fiber at ambient conditions would create a new shape with irreversible plastic deformation (Fig. 5h). However, moisture treatment quickly recovered the original shape. Such a shape memory behavior in both compressive and tensile modes provides many possibilities for the hydrogel microfiber with promising shape-morphing, load-bearing, and actuating applications for soft machines and robots. For example, a twisted and self-balanced hydrogel microfiber with enhanced mechanical strength can be employed as a recoverable rescue rope (Supplementary Fig. 31). A plastic human model (~25 g) tied to the twisted hydrogel fiber slowly descended from a tall building model owing to the high damping capacity of the fiber for reducing shock disturbance (Supplementary Fig. 32). Moisture treatment then fully recovered the length of the rescue rope, enabling repeatable uses for many times.

## Discussion

We here presented a robust hydrogel microfiber that mimics the nanoconfined hierarchical structure of spider silk produced by facile, low-cost, and energy-efficient aqueous pultrusion spinning. The nanoconfinement of the hydrogel microfiber is defined by high-density H-bond clusters embedded in an ionically complexed hygroscopic ductile matrix, which endows the fiber with excellent extensibility, high modulus, crack resistance, ultra-rapid self-healability, moisture sensitivity, damping capacity, and supercontraction. Such a combination of all these intriguing properties as well as spinning sustainability has never been reported in previous artificial thin fibers (see detailed comparison in Supplementary Table 4). In particular, the moisture-induced ultra-rapid and complete crack self-healing is unique to the hydrogel microfiber, which is not even observed in spider silk. We anticipate that the present molecular-level nanoconfinement design in combination with the energy-saving pultrusion spinning procedure represents a considerable step towards artificial smart and robust microfibers for promising uses in miniature soft machines and robots.

## Methods

### Materials

Poly(methacrylic acid) (PMAA, *M*_w_ ≈ 100,000 g mol^−1^, purity ~95–100%, catalog No. 00578) was purchased from Polysciences. Poly(acrylic acid) (PAA, 35 wt% in H_2_O, *M*_w_ ≈ 100,000 g mol^−1^, catalog No. 523925), 2-(dimethylamino)ethylacrylate methyl chloride quarternary salt (DMAEA-Q, 80 wt% in H_2_O, catalog No. 496146), and 3-dimethyl(methacryloyloxyethyl) ammonium propane sulfonate (DMAPS, purity ~95%, catalog No. 537284) were purchased from Sigma-Aldrich. 1-Vinyl-3-ethylimidazolium bromide (ViEt, purity ~99%) was purchased from Meryer Biochemical. 3-Sulfopropyl methacrylate potassium salt (SPMA, purity ~96%) was purchased from Aladdin. 3-(Acrylamidopropyl)trimethyl ammonium chloride (APTMA, 75 wt% in H_2_O) was purchased from Energy Chemical. 2-Methacryloyloxyethyl phosphorylcholine (MPC, purity ~96%) was purchased from Rhawn Reagent. Photoinitiator, 2-ketoglutaric acid (purity ~98%), was purchased from Acros Organics. 8-Anilinonaphthalene-1-sulfonic acid (ANS, purity ~95%) was purchased from Adamas. Unless otherwise stated, all the chemicals were used without further purification. Dragline spider silk (drawn from the major ampullate gland of orb weaver) was kindly provided by Prof. Shengjie Ling of ShanghaiTech University.

### Preparation of PDMAEA-Q/PMAA spinning dope

The optimal spinning solution with 1:2 DMAEA-Q:MAA molar ratio and polymer content of 30 wt% was prepared as follows: 0.3025 g (1.25 mmol) of DMAEA-Q, 0.2148 g (2.5 mmol) of PMAA, and 4.0 mg (2 mol% with respect to DMAEA-Q) of 2-ketoglutaric acid were mixed in 1.06 mL of water. The solution was continuously stirred at room temperature for about 1 h, and then treated with ultrasonication for 10 min to remove bubbles. Photo-induced polymerization (365 nm, 40 W) was performed for 30 min to yield a final uniform and transparent spinning dope. Other spinning dopes with different molar ratios and polymer contents were prepared by adjusting the recipe accordingly. For ANS-doped luminescent hydrogel microfiber, a small amount of 1 wt% ANS (with respect to the total solid content) was added to the spinning dope.

For the preparation of the spinning dopes of PSPMA/PMAA, PAPTMA/PMAA, PViEt/PMAA, PDMAPS/PMAA, and PMPC/PMAA, only the monomer with equal moles was replaced, and the amount of water was adjusted to 1.4, 1.18, 1.2, 1.3, and 1.2 mL, respectively, to optimize the rheological behavior for pultrusion spinning.

### Preparation of PDMAEA-Q/PMAA hydrogel fiber

The as-prepared spinning dope was transferred into a plastic syringe without needle and installed onto a syringe pump. Hydrogel microfibers were directly pulled out of the syringe head with the feeding speed of 200 μL min^−1^ and immediately solidified with the evaporation of excess water at ambient conditions (25 °C, RH 60%). The hydrogel microfibers were collected on a rotating frame with a specified rotation speed (5–25 rpm) and constant parallel moving speed of 4 mm s^−1^. Unless otherwise stated, for all the studies in this paper, the rotation speed was set to 5 rpm, generating hydrogel microfibers with a typical diameter of 125 ± 15 μm.

### Preparation of PDMAEA-Q hydrogel fiber

PDMAEA-Q hydrogel fiber was prepared for control. Considering PDMAEA-Q cannot be easily spun into microfibers, we employed a PTFE tube to shape it. Briefly, 4.0 mg (2 mol%) of 2-ketoglutaric acid was added into 0.3025 g (80 wt% in H_2_O, 1.25 mmol) of DMAEA-Q, and the mixture was stirred for about 10 min to form a transparent solution. The solution was then slowly injected into a PTFE tube (inner diameter: 2 mm). UV-induced polymerization was performed for 30 min, and the hydrogel fiber was finally peeled off from the tube.

### Preparation of PMAA microfiber

0.2148 g of PMAA powder was dissolved in 1.3 mL of water, and the solution was then stirred for about 1 hour to form a spinning dope. Other steps were the same to PDMAEA-Q/PMAA hydrogel microfiber.

### Preparation of PDMAEA-Q/PAA film

0.3025 g (1.25 mmol) of DMAEA-Q, 0.5143 g of PAA (2.5 mmol, 35 wt% solution), and 4.0 mg (2 mol% with respect to DMAEA-Q) of 2-ketoglutaric acid were mixed in 1 mL of water, and continuously stirred for 10 min to achieve a homogeneous solution. The solution was then transferred to a PTFE mold for photo-induced polymerization (365 nm, 40 W) for 30 min to obtain a highly viscous solution. The solution was left in air to let excess water evaporate at ambient conditions (25 °C, RH 60%), and PDMAEA-Q/PAA film was finally obtained with moisture equilibrium.

### Characterizations

The rheological behavior of the spinning dope was investigated on a HAAKE MARS 60 rheometer. Dynamic frequency sweep was measured from 1 to 100 rad/s at 25 °C in the oscillation mode, and the oscillatory stress was set to 300 Pa. SEM and elemental mapping images were taken using an environmental scanning electron microscope (Quanta 250, Czech Republic). Attenuated total reflection Fourier transform infrared (ATR-FTIR) spectra were taken on a Nicolet iS50 (Thermo Fisher Scientific) spectrometer with a diamond ATR crystal as the window material. Synchrotron FTIR spectra were collected at the BL01B beamline in the Shanghai Synchrotron Radiation Facility (SSRF) with Nicolet 6700 Fourier transform infrared spectrometer. Optical images of hydrogel microfibers were taken on a stereoscopic microscope (Olympus SZX7). The glass transition temperatures were measured on a differential scanning calorimeter (DSC, Q250, TA Instruments) with a heating rate of 50 °C min^−1^ under nitrogen flow. For humidity control to equilibrate the hydrogel microfiber, the relative humidities of 10%, 30%, 55%, 75% and 85% were realized by the supersaturated solutions of LiCl, MgCl_2_, NaBr, NaCl and KCl, respectively. TEM image was taken on a transmission electron microscope (JEM-2100). AFM height and phase images were scanned from an atomic force microscope (MFP-3D Bio). Contact angles were tested on a contact angle goniometer (XG-CAMC3, Shanghai Xuanyi-Guangxi, China). Water contents and hydration numbers at varied humidities were measured by equilibrating the fiber in a constant climate chamber (Binder KMF115) at fixed humidities for 3 hours. The transmittance of the PDMAEA-Q/PMAA film was measured on a UV-Vis-NIR spectrometer (Shimadzu UV-2600). The size distributions of polymer solutions were determined by dynamic light scattering (DLS, Malvern Zetasizer Nano ZS). ^1^H NMR spectra were recorded on Bruker AV-400 using D_2_O as the solvent. Polarized optical observations were performed on a polarizing optical microscope (POM, Olympus BX53-P) with a 530 nm tint plate.

### Small-angle X-ray scattering (SAXS)

SAXS experiments were performed at the SSRF beamline BL16B at an X-ray energy of 10.0 keV, which corresponds to a wavelength of λ = 1.24 Å. PDMAEA-Q/PMAA hydrogel microfiber was measured perpendicular to the beam with a sample-detector distance of 1.87 m to cover the scattering vector *q* range from 0.1 to 4 nm^−1^ (*q* is the scattering vector, *q* = (4π/λ)sinθ, and 2*θ* the scattering angle). The scattering patterns were obtained with a short exposure time (60 s), and the air background was subtracted. The SAXS pattern was azimuthally and radially averaged to obtain the 1D intensity profile. The 1D SAXS scattering curve was fitted by the 2-level Beaucage model^54^.

### Tensile tests

General tensile tests were carried out on a vertical dynamometer (ESM303, MARK-10) at room temperature (25 °C) and RH 60%. The environmental humidity was controlled by a humidifier. Unless otherwise stated in this paper, the strain rate was fixed to 0.02 s^−1^. The average diameter was obtained from five measurements along the fiber using an optical microscope.

### Self-healing tests

The hydrogel microfiber was placed horizontally on a glass substrate, and multiple cracks were introduced with a scalpel under a microscope. For healing, a humidifier was placed perpendicular to the microfiber to apply moisture stimulation for 10, 20, and 30 s, respectively. The repaired hydrogel microfiber was then equilibrated at ambient conditions (25 °C, RH 60%) for 10 min before mechanical tests.

### Calculation of activation volume and energy

The activation volume and energy were calculated from the Eyring model^43^

1 $${\sigma }_{{{{{{\rm{y}}}}}}} \,\approx\, \frac{2{kT}}{{{V}}_{{{{{{\rm{a}}}}}}}}\,{{{{{\mathrm{ln}}}}}}\,\dot{{{{{{\rm{\varepsilon}}}}}} }+\frac{2{{E}}_{{{{{{\rm{a}}}}}}}}{{{V}}_{{{{{{\rm{a}}}}}}}}$$

where *V*_a_ is the activation volume, *E*_a_ the activation energy, *ε̇* the strain rate, and *σ*_y_ the yield stress.

### Calculation of energy density

The energy density was calculated by using the work done by the hydrogel microfiber divided by the microfiber’s drying mass.

### Calculation of damping capacity

The damping capacity was calculated by the ratio of the dissipated energy to the stored energy in the tensile curves. The stored energy is the integral area under the stress-strain curve upon loading. The dissipated energy is calculated by subtracting the integral area upon unloading from the stored energy.

### Calculation of fracture energy

Considering the cylinder shape of microfibers, the fracture energy was tentatively calculated from pure shear test^48^. Two different fibers, notched and unnotched, were used. The unnotched fiber was pulled to measure the stress-strain curve. The notched fiber was prepared using a scalpel to cut a notch (depth ~30 μm for hydrogel microfiber). Fracture energy (*Γ*) was calculated from the integral area under the stress–strain curve of unnotched fiber with the initial clamp distance (*H*), and the formula is $\varGamma=H{\int }_{0}^{{\varepsilon }_{c}}\sigma {{{{{\rm{d}}}}}}\varepsilon$, where *ε*_c_ is the critical strain at which fracture of notched fiber occurred.

### Calculation of crosslinking densities

The effective crosslinking density (*v*_e_) was determined with the classical rubber elasticity theory proposed by Flory^55^. The equation is given by

2 $${G \,\approx\, {RTv}e\varPhi }^{1/3}$$

where, *R* is the gas constant, *T* the absolute temperature (= 298.15 K), *Φ* the volume fraction (could be roughly determined by weight fraction; for hydrogel microfiber, *Φ* = 0.69 at RH 60%). The modulus, *G*, is the slope of nominal stress and (*λ*-*λ*^−2^) (*λ* is the deformation ratio; *λ* = strain +1).

### Molecular dynamics simulation

The interaction energies of the studied pairs were calculated from molecular dynamics simulation by the software Materials Studio, ver. 2019. The geometrical optimization was performed in the DMol3 module at NVE conditions for 5000 steps. The interaction energy was calculated by subtracting the added potential energies of all the individual components from the whole potential energy.

### Temperature-variable FTIR measurement

For sample preparation, one drop of spinning dope was cast on a CaF_2_ tablet and scratched to a film with suitable thickness. Excess water was then allowed to evaporate at room temperature to obtain a film, which was further incubated in a chamber at RH 60% for 12 h to reach moisture equilibrium. Afterwards, the sample was covered with another CaF_2_ tablet and sealed for temperature-controlling measurement. The sealed sample was heated from 25 to 70 °C with an interval of 5 °C in the transmission mode on a Nicolet iS50 FTIR spectrometer.

### 2D correlation spectroscopy (2DCOS)

All the temperature-dependent FTIR spectra of hydrated PDMAEA-Q/PMAA film from 25 to 70 °C were used for performing 2DCOS analysis. 2DCOS analysis was carried out using the software, 2D Shige ver. 1.3 (©Shigeaki Morita, Kwansei-Gakuin University, Japan, 2004-2005), and was further plotted into the contour maps by the Origin program, ver. 2021. In the contour maps, red colors are defined as positive intensities, while blue colors as negative ones.

### Statistics and reproducibility

All experiments were repeated independently with similar results for at least three times.

## Supplementary information

Supplementary Information

Description of Additional Supplementary Files

Supplementary Movie 1

Supplementary Movie 2

Supplementary Movie 3

Supplementary Movie 4

Supplementary Movie 5

## Data Availability

All data supporting the findings of this study are available within this article and Supplementary Information or from the corresponding author upon request. The data generated in this study are provided in the Supplementary Information/Source Data file. Source data are provided with this paper.

## Source data

Source Data
