# Supplementary material for: Aqueous spinning of robust, self-healable, and crack-resistant hydrogel microfibers enabled by hydrogen bond nanoconfinement
Source: Nat Commun. 2023 Mar 13;14:1370. doi: 10.1038/s41467-023-37036-4 (PMC10011413; doi:10.1038/s41467-023-37036-4)
Supplement: Supplementary file 1 — Supplementary Information [file 41467_2023_37036_MOESM1_ESM.pdf]

Supplementary Information

Aqueous spinning of robust, self-healable, and crack-resistant hydrogel microfibers enabled by hydrogen bond nanoconfinement

Yingkun Shi[1], Baohu Wu[2], Shengtong Sun[1,*] and Peiyi Wu[1,*]

[1]State Key Laboratory for Modification of Chemical Fibers and Polymer Materials, College of Chemistry and Chemical Engineering & Center for Advanced Low-dimension Materials, Donghua University, Shanghai 201620, China

[2]Jülich Centre for Neutron Science (JCNS) at Heinz Maier-Leibnitz Zentrum (MLZ) Forschungszentrum Jülich, Garching 85748, Germany

Correspondence should be addressed to S.T.S. (email: shengtongsun@dhu.edu.cn) and P.Y.W. (email: wupeiyi@dhu.edu.cn)

This file includes

Extended Methods

Suppl. Fig. 1. Schematic process for preparing hydrogel microfibers

Suppl. Fig. 2. Rheological behavior of the spinning dopes with different polymer contents

Suppl. Fig. 3. Collected PDMAEA-Q/PMAA hydrogel microfibers on a rotating frame

Suppl. Fig. 4. UV-vis spectrum of PDMAEA-Q/PMAA film

Suppl. Fig. 5. EDS mapping images of dried PDMAEA-Q/PMAA hydrogel microfiber

Suppl. Fig. 6. Synchrotron IR spectra of PDMAEA-Q/PMAA hydrogel microfiber

Suppl. Fig. 7. Water contact angles of PDMAEA-Q/PMAA and PDMAEA-Q/PAA films

Suppl. Fig. 8. Water stability of PDMAEA-Q/PMAA and PDMAEA-Q/PAA films

Suppl. Fig. 9. Tensile stress-strain curve of PDMAEA-Q/PAA film

Suppl. Fig. 10. Photos and tensile curves of PAPTMA/PMAA and PViEt/PMAA microfibers

Suppl. Fig. 11. Photos and tensile curves of PSPMA/PMAA microfiber

Suppl. Fig. 12. Photos of polymerizing zwitterionic monomers in the presence of PMAA

Suppl. Fig. 13. AFM height images of pure PDMAEA-Q and PMAA films

Suppl. Fig. 14. XRD profile of PDMAEA-Q/PMAA film

Suppl. Fig. 15. SAXS scattering curve of the spinning dope

Suppl. Fig. 16. ATR-FTIR spectral comparison of PMAA film equilibrated at different RHs

Suppl. Fig. 17. Calculated interaction energies from molecular dynamics simulation

Suppl. Fig. 18. Tensile curves of hydrogel microfibers in three batches

Suppl. Fig. 19. Tensile curves of hydrogel microfibers with varying molar ratios

Suppl. Fig. 20. Tensile curves of dragline spider silk with/without notch
Suppl. Fig. 21. True stress-strain curves of hydrogel microfiber at RH 80% and 90%
Suppl. Fig. 22. Stability tests of hydrogel microfiber in water and HCl solution
Suppl. Fig. 23. Moisture sensitivities of hydrogel microfiber and spider silk
Suppl. Fig. 24. Ionic conductivities of hydrogel microfiber at different RHs
Suppl. Fig. 25. Moisture-induced self-healing process of pure PDMAEA-Q and PMAA fibers
Suppl. Fig. 26. DSC curves of hydrogel microfibers with varying molar ratios and humidities
Suppl. Fig. 27. Loading-unloading curves of hydrogel microfiber with/without moisture treatment
Suppl. Fig. 28. SEM images of hydrogel microfiber before and after supercontraction
Suppl. Fig. 29. POM images of hydrogel microfiber in the whole supercontraction process
Suppl. Fig. 30. Photos for measuring load-dependent supercontraction ratios
Suppl. Fig. 31. Tensile curves and SEM images of original and twisted hydrogel microfibers
Suppl. Fig. 32. Photos of the twisted hydrogel microfiber as the rescue rope

Suppl. Table 1. Comparison of the combinatory properties of spider silk and PDMAEA-Q/PMAA hydrogel microfiber
Suppl. Table 2. Signs of the main cross-peaks in 2DCOS synchronous and asynchronous spectra
Suppl. Table 3. Comparison of the fracture energies and elongations among PDMAEA-Q/PMAA hydrogel microfiber and other materials
Suppl. Table 4. Comparison of the properties and preparing conditions among typical artificial microfibers

References S1-S63

Extended Methods

2D correlation spectroscopy (2DCOS)

2DCOS, as a mathematical method, is very suitable for the investigation of the variations of chemical groups. Its basic principle was first proposed by Noda and has been applied more and more widely to follow spectral variations under various external perturbations, such as time, temperature, pressure, concentration, and other physical variables[S1]. By spreading the original spectral information along a second dimension, spectral resolution enhancement can be achieved, allowing the additional important information about molecular motions or conformational changes not readily visible in conventional analysis to be extracted.

2DCOS includes two types of correlation maps, the synchronous spectrum, which reflects simultaneous changes between two wavenumbers, and the asynchronous spectrum, which significantly enhances the spectral resolution. The auto-peaks only appear along the diagonal in the synchronous spectrum, while the cross-peaks can appear in both synchronous and asynchronous spectra. The judging rule of the sequence can be summarized as Noda's rule-that is, if the multiplication of the signs of cross-peaks (ν_1, ν_2, and assume $\nu_1 > \nu_2$) in synchronous and asynchronous spectra is positive, the change at ν_1 may occur before ν_2, and vice versa[S2].

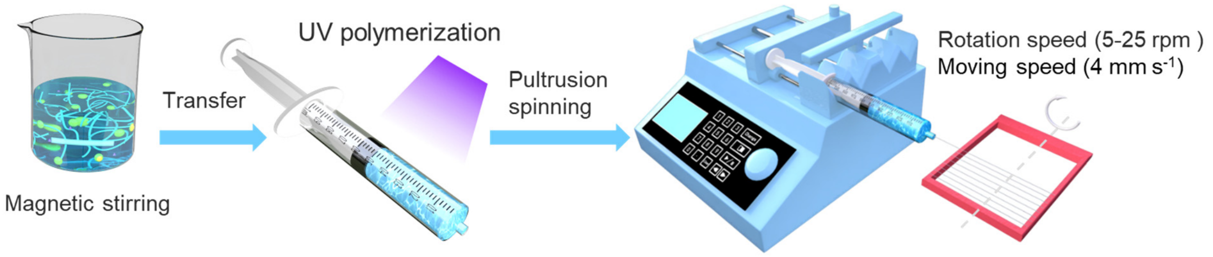

Supplementary Fig. 1. Schematic process for preparing PDMAEA-Q/PMAA hydrogel microfibers. The initial precursor contains PMAA, DMAEA-Q, and photoinitiator.

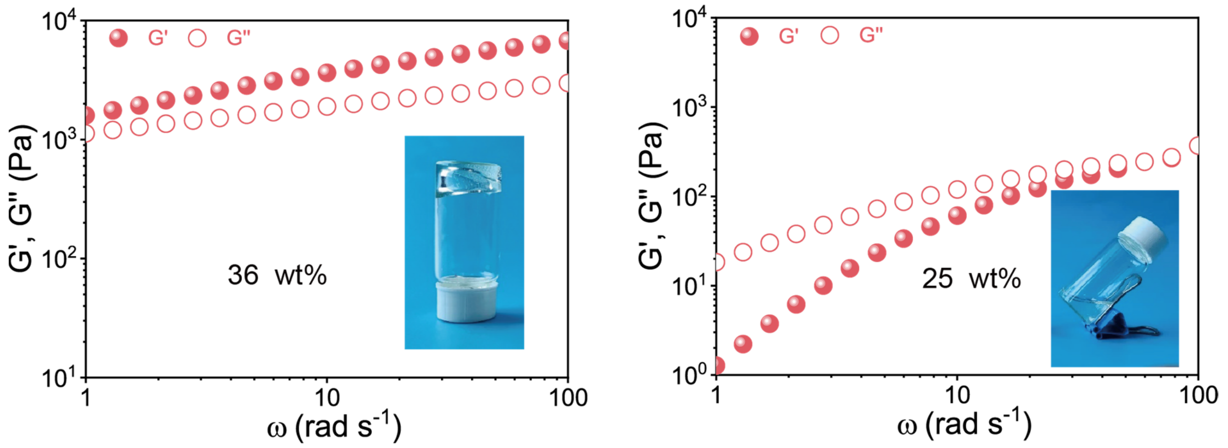

Supplementary Fig. 2. Rheological behavior of the spinning dopes with the polymer contents of 36 wt% and 25 wt%, respectively. The 25 wt% dope appeared as a dilute liquid with the sol-gel relaxation time of ~0.14 s, while the 36 wt% dope appeared as a gel with a longer relaxation time than 6 s. Both these two concentrations are unsuitable for continuous pultrusion spinning.

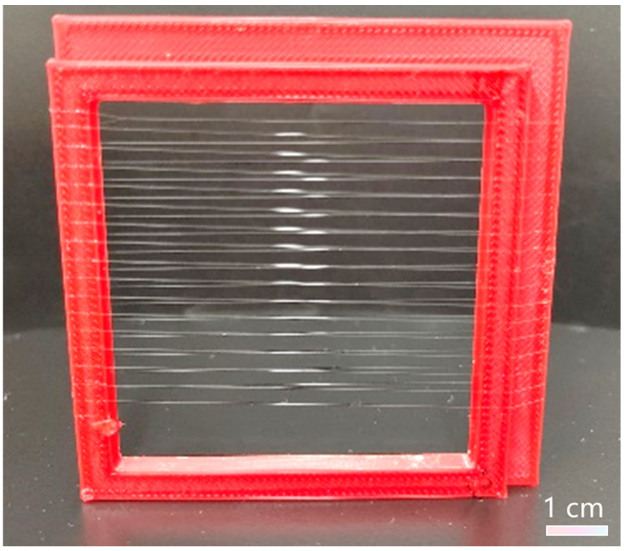

Supplementary Fig. 3. Collected PDMAEA-Q/PMAA hydrogel microfibers on a rotating frame.

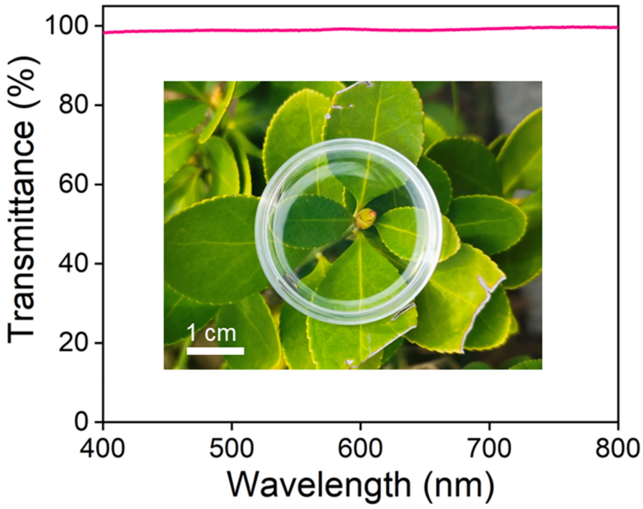

Supplementary Fig. 4. UV-vis spectrum of PDMAEA-Q/PMAA film with the same composition to hydrogel microfiber. The film thickness is 1 mm. Photo credit: Yingkun Shi, Donghua Univerity.

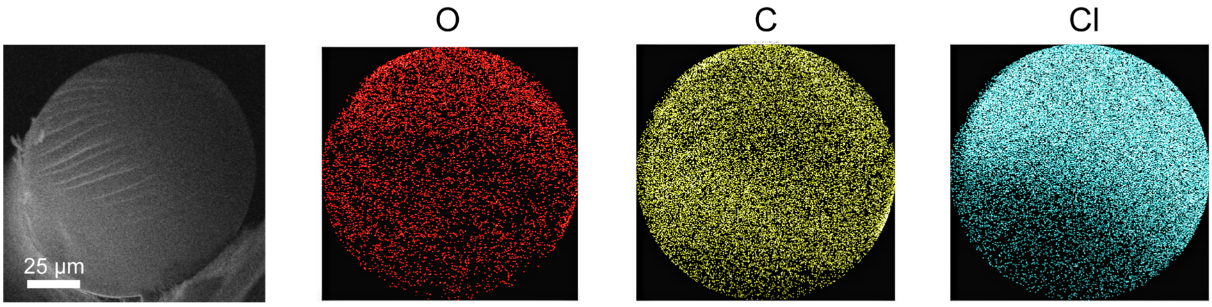

Supplementary Fig. 5. EDS mapping images of hydrogel microfiber. O, C, and Cl elements in the cross-section of dried PDMAEA-Q/PMAA hydrogel microfiber.

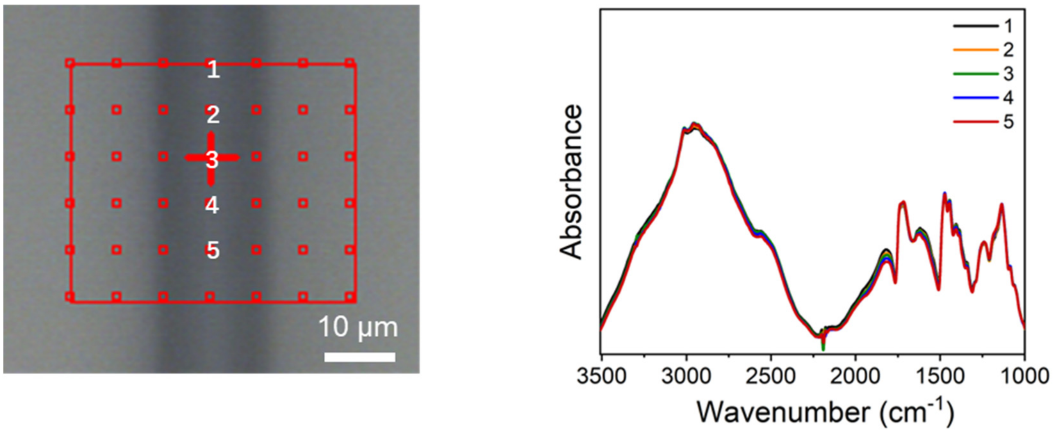

Supplementary Fig. 6. Synchrotron IR spectra of PDMAEA-Q/PMAA hydrogel microfiber at different positions. The almost identical IR spectra suggest the structural homogeneity of the microfiber at the macroscopic scale. The diameter of hydrogel microfiber is ~15 μm to meet the sample thickness requirement for transmission IR measurement.

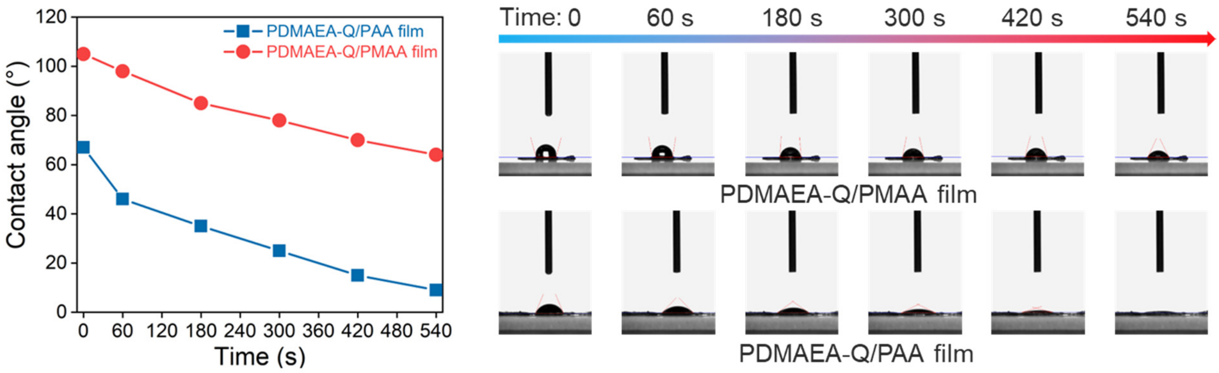

Supplementary Fig. 7. Time-dependent water contact angle changes of PDMAEA-Q/PMAA and PDMAEA-Q/PAA films. Owing to the presence of α-methyl group in PMAA, PDMAEA-Q/PMAA film is more hydrophobic than PDMAEA-Q/PAA. The molar ratios of DMAEA-Q:MAA and DMAEA-Q:AA are both 1:2. As time extended, water molecules penetrated into the films leading to the gradual attenuation of contact angles.

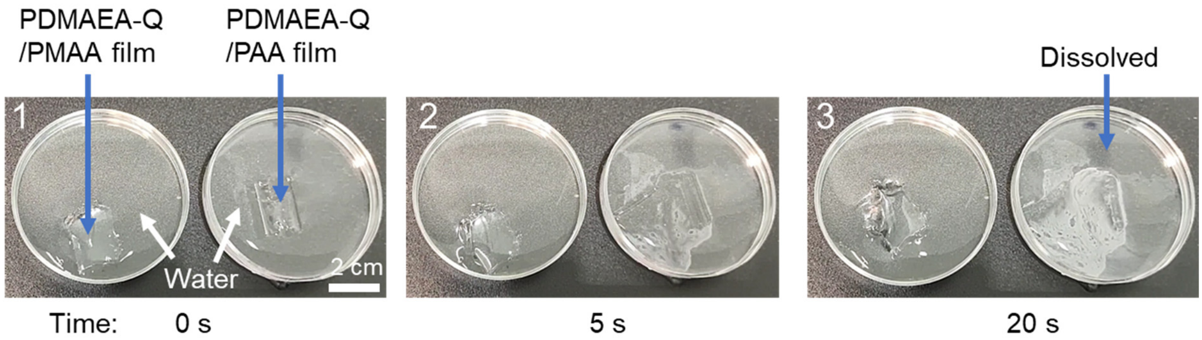

Supplementary Fig. 8. Stability test of PDMAEA-Q/PMAA and PDMAEA-Q/PAA films by immersing in water. PDMAEA-Q/PMAA film remained stable in water, while PDMAEA-Q/PAA film readily dissolved in 20 s.

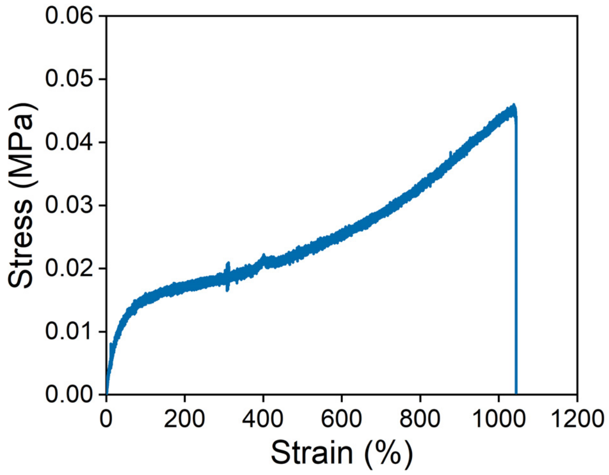

Supplementary Fig. 9. Tensile stress-strain curve of PDMAEA-Q/PAA film (RH 60%; strain rate: 0.02 s^{-1}).

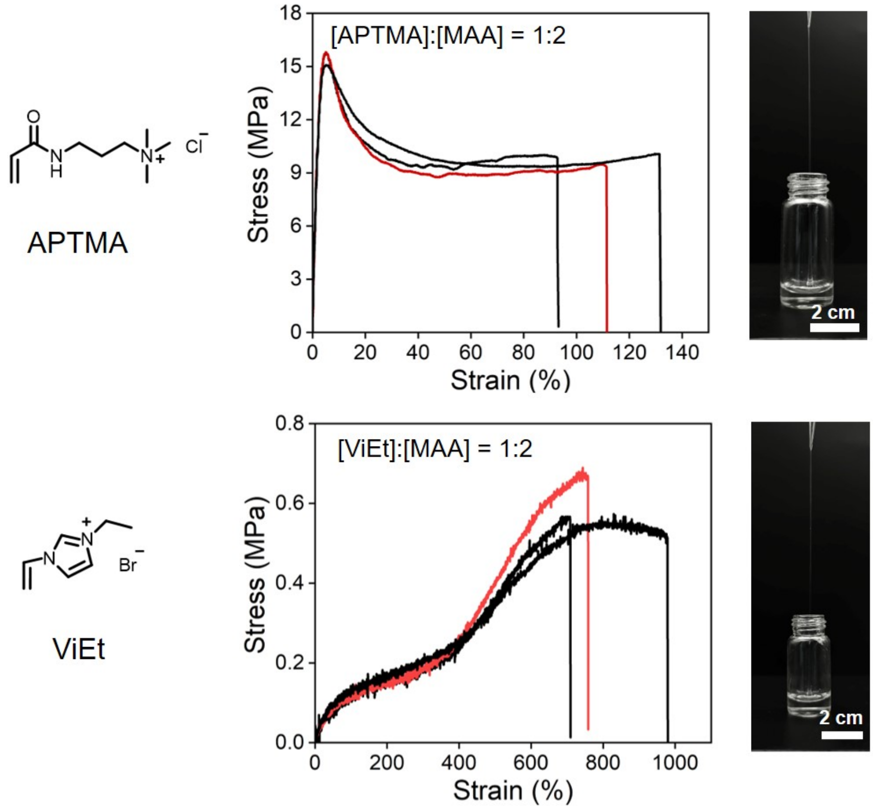

Supplementary Fig. 10. Photos and tensile curves of PAPTMA/PMAA and PViEt/PMAA hydrogel microfibers (RH 60%; strain rate: 0.02 s^{-1}). The molar ratios of [APTMA]:[MAA] and [ViEt]:[MAA] were both fixed to 1:2, similar to PDMAEA-Q/PMAA. Both the two fibers were tested for three times. Using positively charged monomers could generate extensile hydrogel microfibers with good spinnability. In particular, the acrylamide-type PAPTMA could afford hydrogel microfibers as tough as acrylate-type PDMAEA-Q/PMAA, while the strongly hygroscopic PViEt generated soft yet highly stretchable hydrogel microfibers.

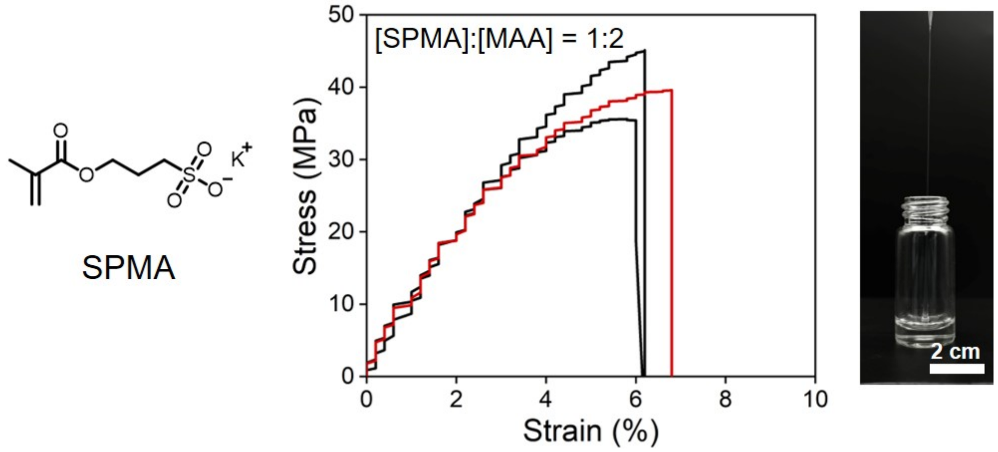

Supplementary Fig. 11. Photos and tensile curves of PSPMA/PMAA microfiber (RH 60%; strain rate: 0.02 s^{-1}). The molar ratio of [SPMA]:[MAA] was fixed to 1:2. The fiber was tested for three times. It is noted that, the negatively charged PSPMA is not able to break the strong self-associated H-bonds of PMAA, resulting in stiff yet brittle fibers.

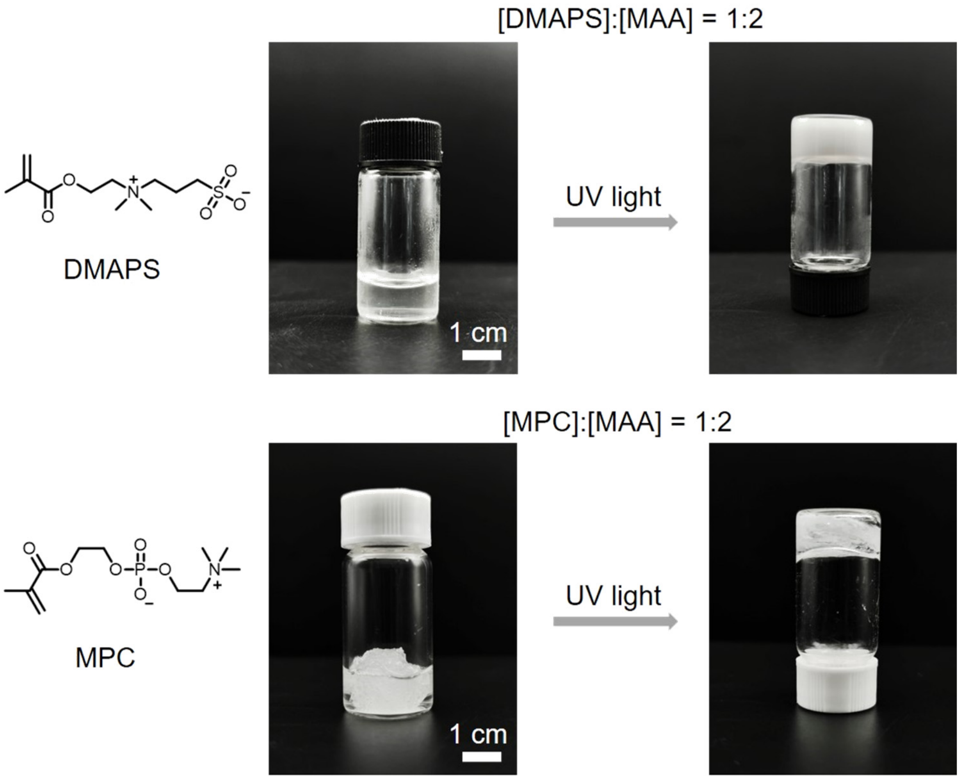

Supplementary Fig. 12. Photos of polymerizing zwitterionic monomers in the presence of PMAA. The molar ratios of [DMAPS]:[MAA] and [MPC]:[MAA] were both fixed to 1:2. Polymerizing zwitterionic monomers in the presence of PMAA led to gels or precipitates and thus no spinnability. This may be caused by the strong self-association of polyzwitterions that additionally act as crosslinkers for PMAA chains.

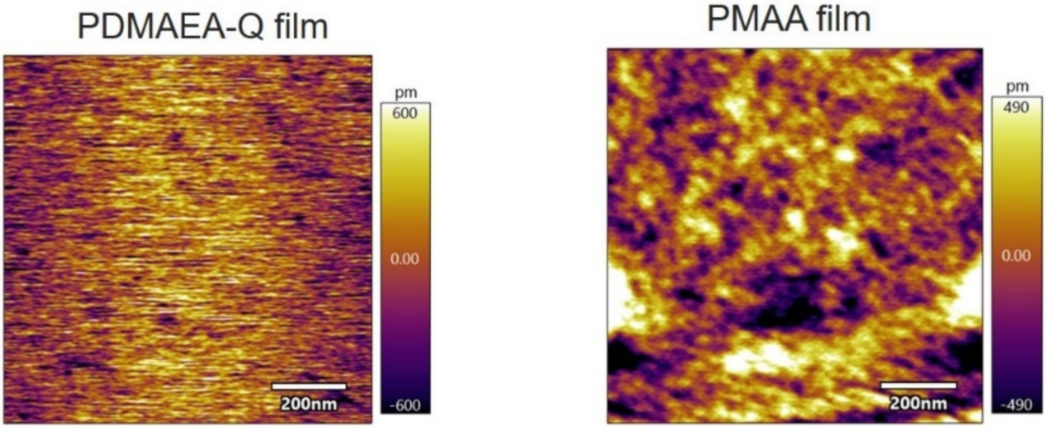

Supplementary Fig. 13. AFM height images of pure PDMAEA-Q and PMAA films. The PDMAEA-Q film is homogenous with no apparent phase separation. However, PMAA forms numerous H-bond clusters with a mean size of 14.5 nm.

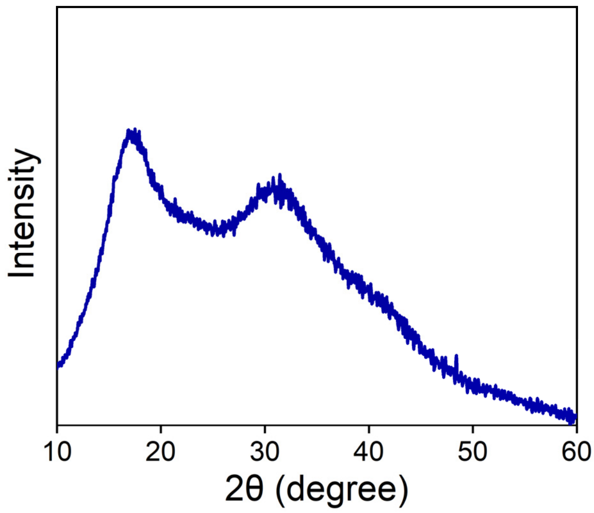

Supplementary Fig. 14. X-ray diffraction (XRD) profile of PDMAEA-Q/PMAA film. No sharp diffraction peaks are observed indicating its amorphous structure. However, the appearance of narrow peaks indicates that short-range order may exist due to the presence of H-bond clusters.

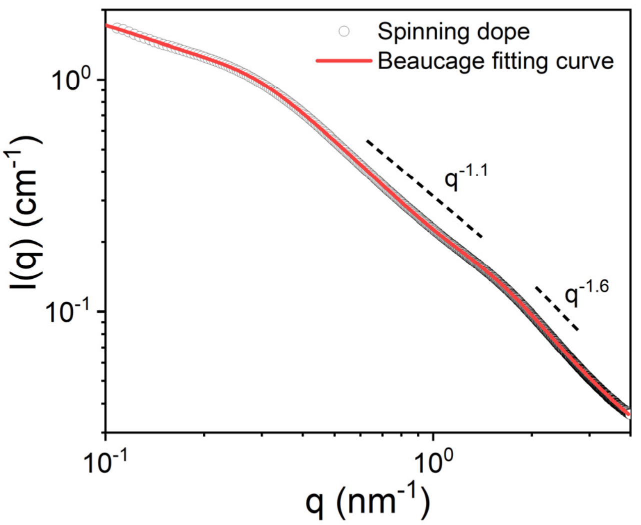

Supplementary Fig. 15. SAXS scattering curve of the spinning dope. The two-level Beaucage fitting suggests the presence of nanofibrillar structures consisting of primary particles (size ~6.5 nm) and second particles (size ~30 nm). The scattering curve in the high q range (>1.5 nm^{-1}) with a power law slope of -1.6 indicates the swollen chain-like molecular structure. The scattering curve in the intermediate q range (0.3 - 1.5 nm^{-1}) with a power law slope of -1.1 reveals the presence of rigid rod-like structure, which is assembled from the primary chain-like structures.

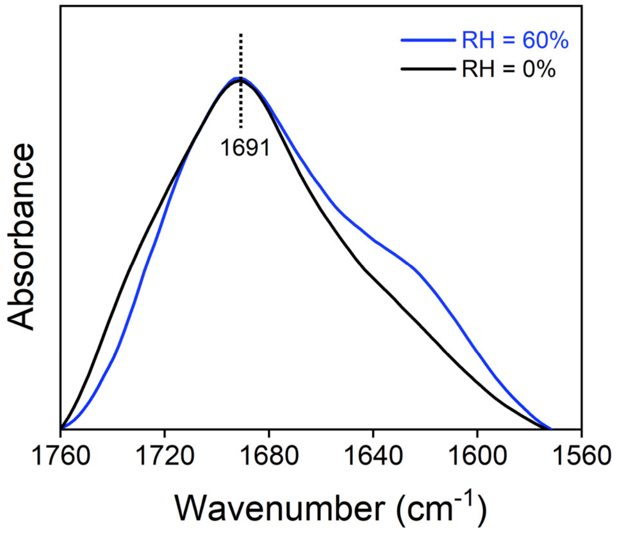

Supplementary Fig. 16. ATR-FTIR spectral comparison of PMAA film equilibrated at RH 0% and 60%, respectively. Due to the stabilizing effect of hydrophobic α-methyl groups, the characteristic *ν*(COOH) at 1691 cm^{-1} assigned to H-bond clusters is almost unchanged with increasing humidity.

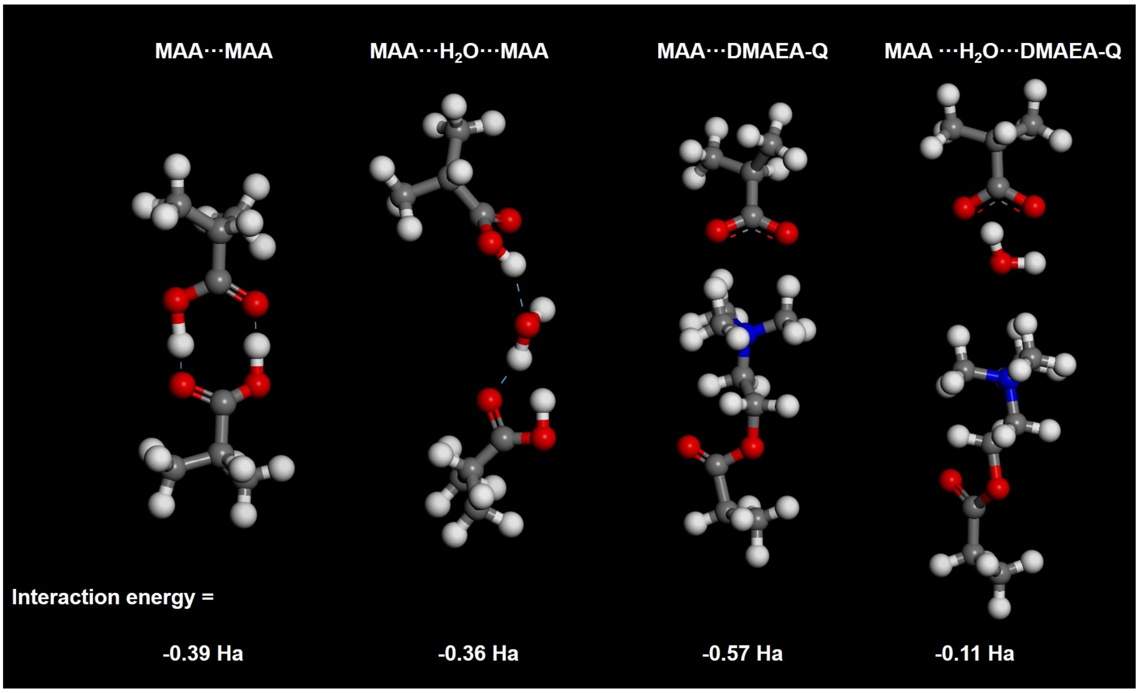

Supplementary Fig. 17. Calculated interaction energies of four interaction pairs from molecular dynamics simulation (Ha = Hartree). For simulation, saturated model molecules for the two monomers were employed to represent the real molecular state of polymers more precisely. The grey, red, blue, and white balls represent C, O, N, and H atoms, respectively.

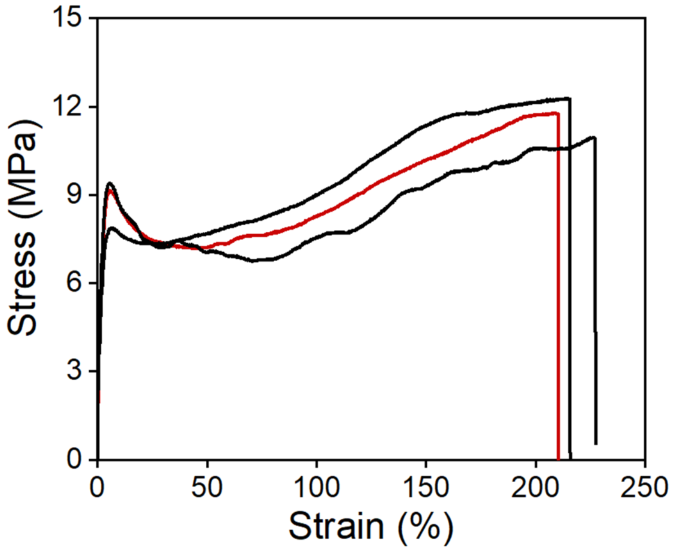

Supplementary Fig. 18. Tensile curves of PDMAEA-Q/PMAA hydrogel microfibers in three batches (strain rate: 0.02 s^{-1}). The tensile behavior of hydrogel microfiber is highly reproducible.

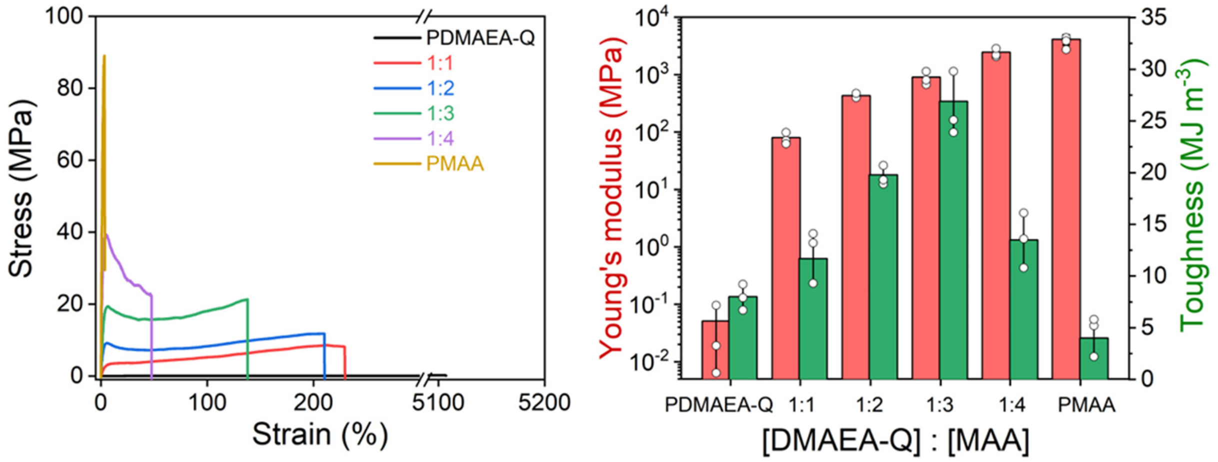

Supplementary Fig. 19. Tensile curves of PDMAEA-Q/PMAA hydrogel microfibers with varying DMAEA-Q:MAA molar ratios (RH 60%; strain rate: 0.02 s^{-1}). The pure PDMAEA-Q fiber, produced in a PTFE tube, can be stretched up to 5107% with a rather low modulus of 51 kPa. The pure PMAA microfiber is rather stiff with a high Young′s modulus of 4.1 GPa yet very small elongation of 5.5%. The PDMAEA-Q/PMAA hydrogel microfibers are both extensible and tough, and with increasing DMAEA-Q:MAA molar ratios, showed reduced Young′s moduli and increased elongations. Young′s modulus and toughness data are presented as mean values ± SD, n = 3 independent samples.

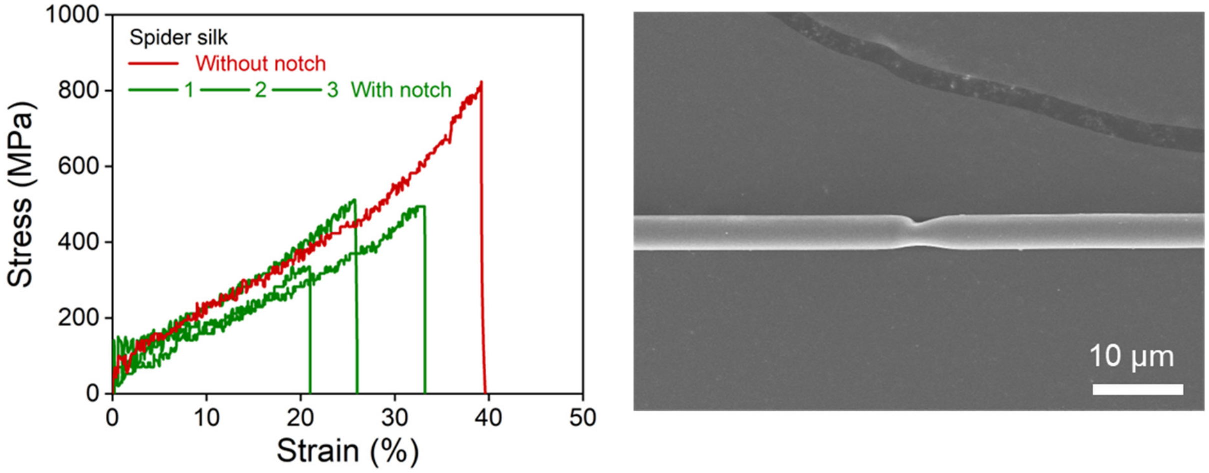

Supplementary Fig. 20. Tensile curves of dragline spider silk with/without notch (RH 60%; strain rate: 0.02 s^{-1}). The SEM image shows a notched spider silk microfiber (diameter ~3.8 μm; notch depth ~1 μm). The critical strain was selected to be the average fracture strain of notched fibers in three batches. The fracture energy of dragline spider silk was calculated to be 735 kJ m^{-2}.

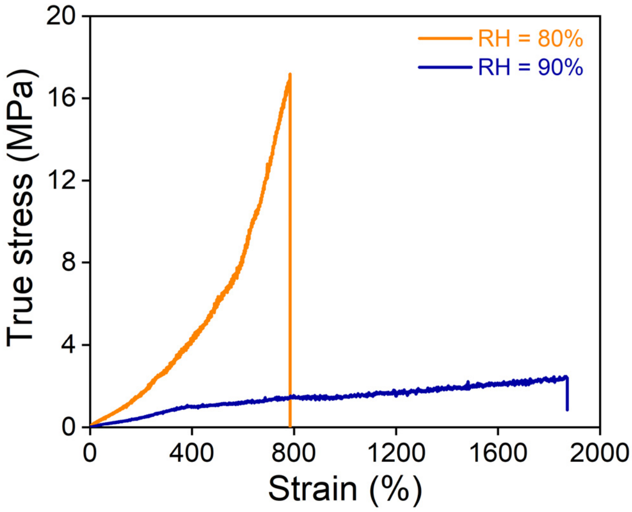

Supplementary Fig. 21. True stress-strain curves of PDMAEA-Q/PMAA hydrogel microfiber at RH 80% and 90%, respectively. The J-shaped curve at RH 80% indicates a typical strain-stiffening behavior, which suggests that the H-bond clusters remained stable serving as strong crosslinks at large strains. Further increasing humidity to RH 90% caused the diminishing of strain-stiffening behavior, suggesting water can still plasticize H-bond clusters at enough high humidities.

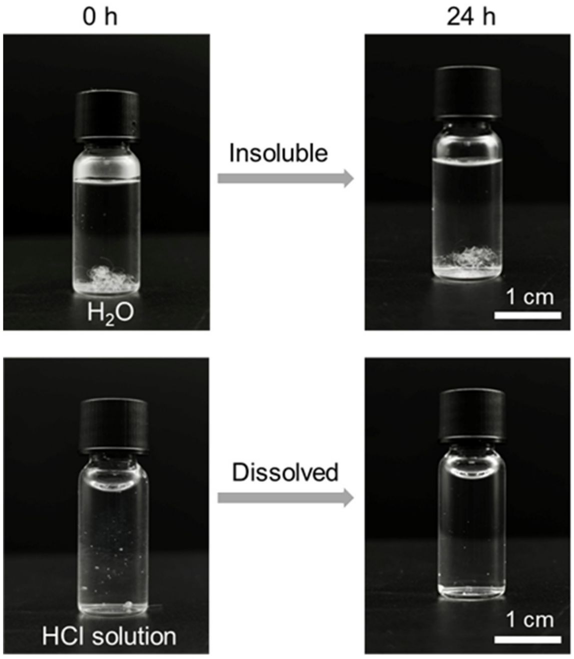

Supplementary Fig. 22. Stability tests of PDMAEA-Q/PMAA hydrogel microfiber in water and HCl solution for 24 h. With the diffusion of formed HCl into water, the hydrogel microfiber remained stable due to enhanced PMAA-related physical crosslinks. As a comparison, the hydrogel microfiber can be fully dissolved in HCl solution (pH ~2.5) to recover the initial spinning sol state.

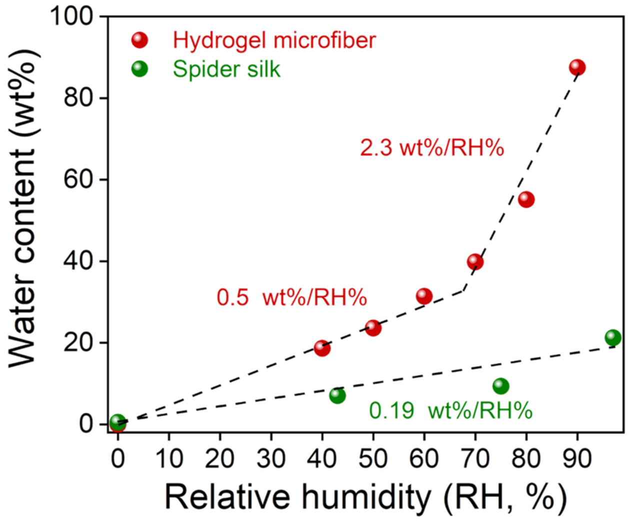

Supplementary Fig. 23. Humidity-dependent water content changes of PDMAEA-Q/PMAA hydrogel microfiber and spider silk. The water content data for spider silk were cited from literature.[S3] As shown, PDMAEA-Q/PMAA hydrogel microfiber has both higher water contents and moisture sensitivities than spider silk in the whole humidity range.

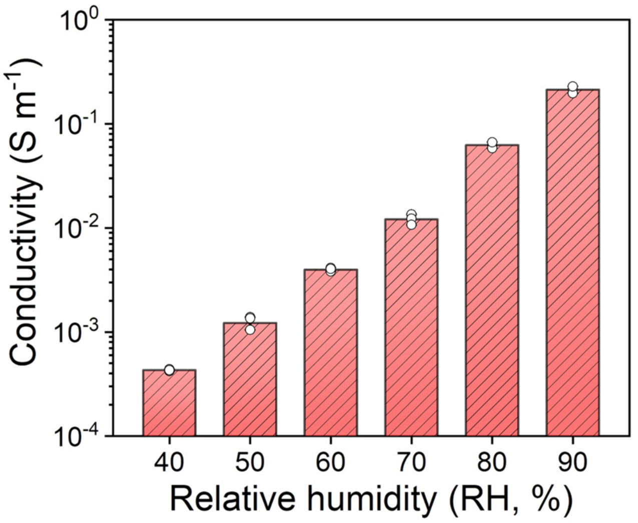

Supplementary Fig. 24. Ionic conductivities of PDMAEA-Q/PMAA hydrogel microfiber at different humidities. Conductivity data are presented as mean values ± SD, n = 3 independent samples.

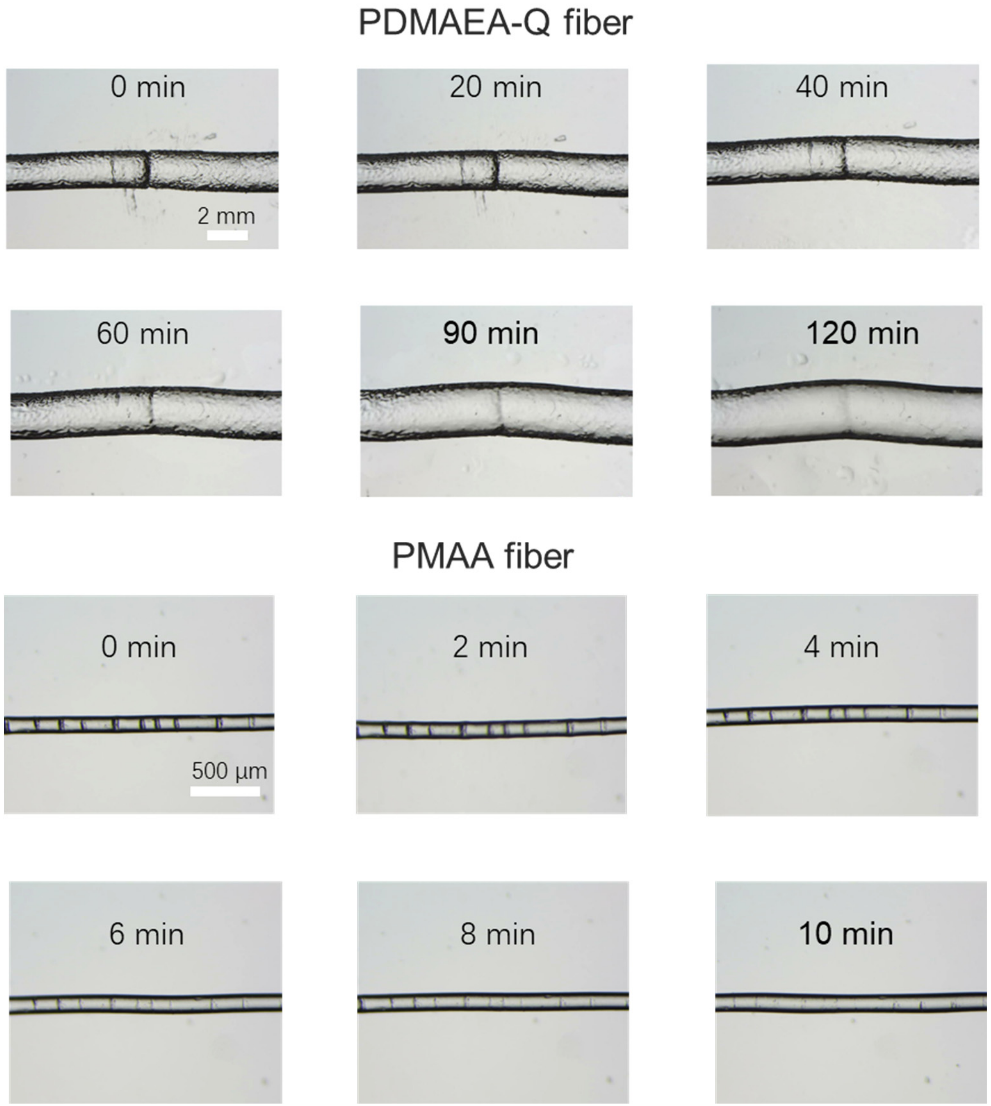

Supplementary Fig. 25. Moisture-induced self-healing processes of pure PDMAEA-Q and PMAA fibers.

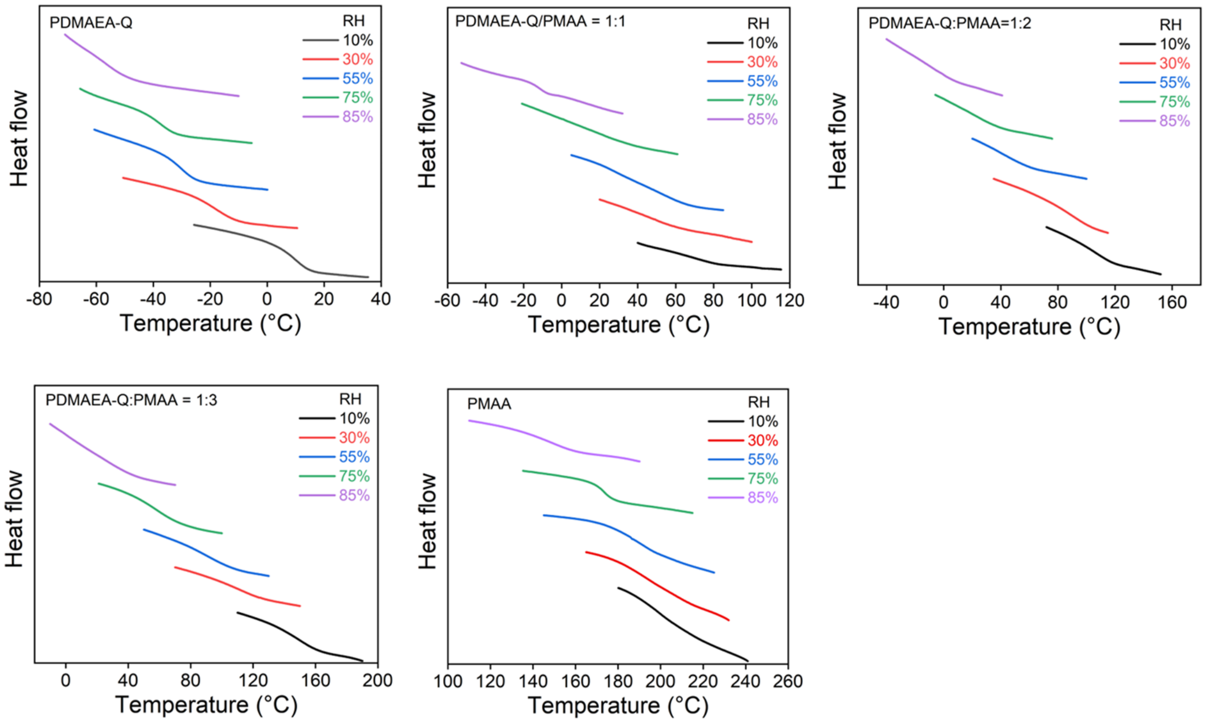

Supplementary Fig. 26. DSC heating curves of PDMAEA-Q/PMAA hydrogel microfibers with varying molar ratios and humidities. The statistical plot of the measured glass transition temperatures is shown in Fig. 5a.

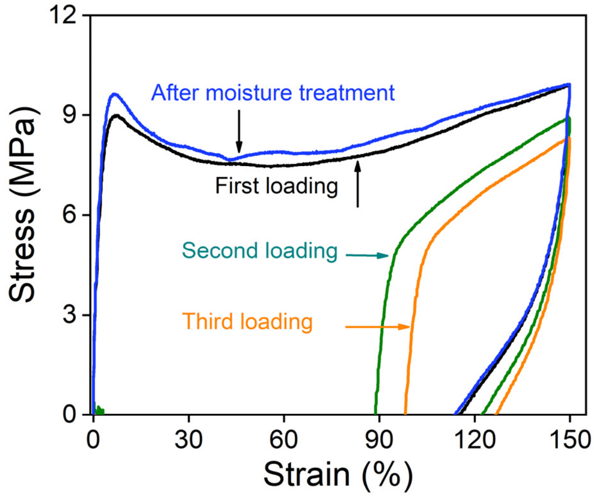

Supplementary Fig. 27. Loading-unloading curves of PDMAEA-Q/PMAA hydrogel microfiber with and without moisture treatment. The almost coincidental curves before and after treatment reveal the totally reversible supercontraction properties of the microfiber.

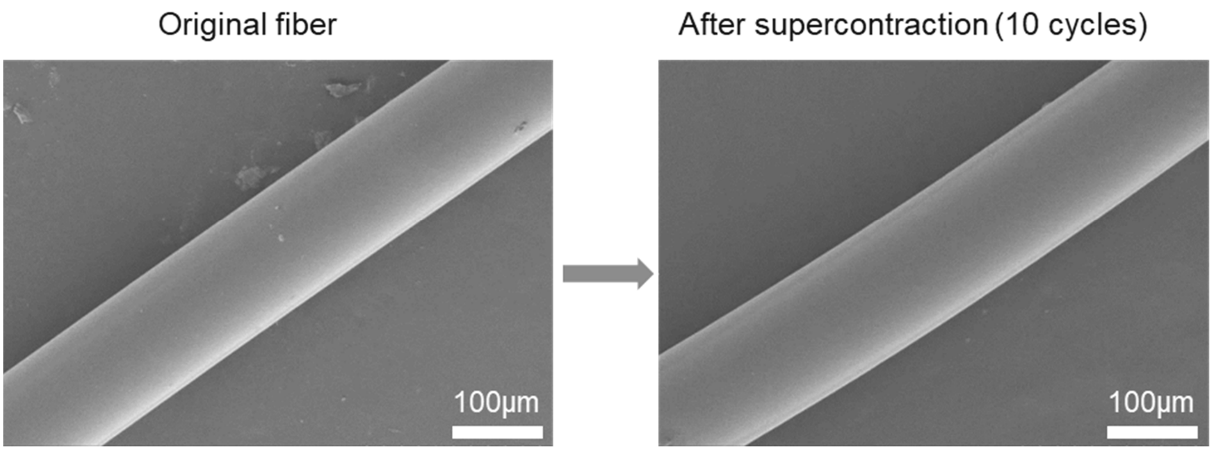

Supplementary Fig. 28. SEM images of PDMAEA-Q/PMAA hydrogel microfiber before and after supercontraction for 10 cycles. Almost no changes of fiber diameter were observed.

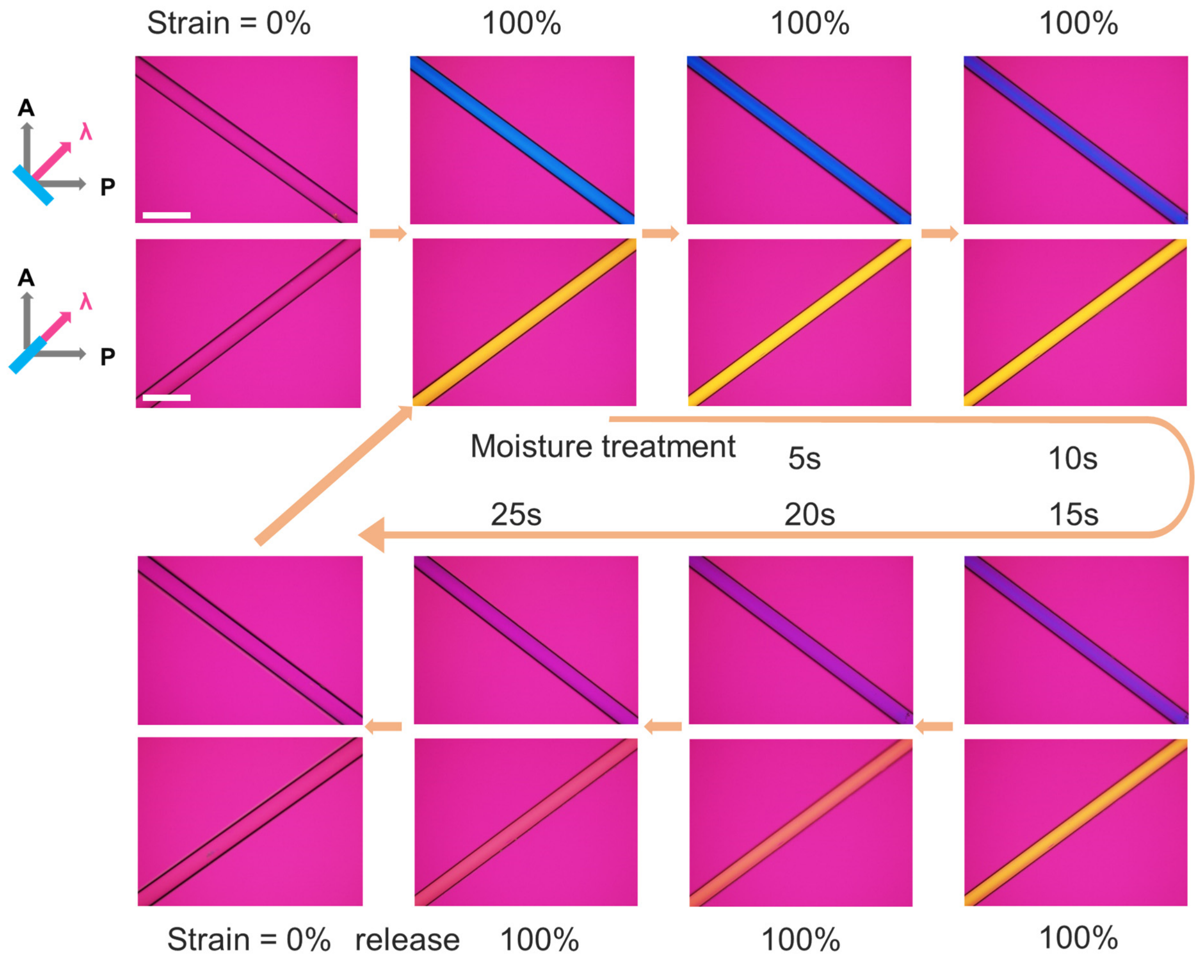

Supplementary Fig. 29. POM images of PDMAEA-Q/PMAA hydrogel microfiber in the whole supercontraction process. Scale bar = 300 μm. All the images were taken in the presence of 530 nm tint plate at the azimuth angles of -45° and 45°, respectively. The stretch-induced structural orientation can be fully eliminated by moisture treatment.

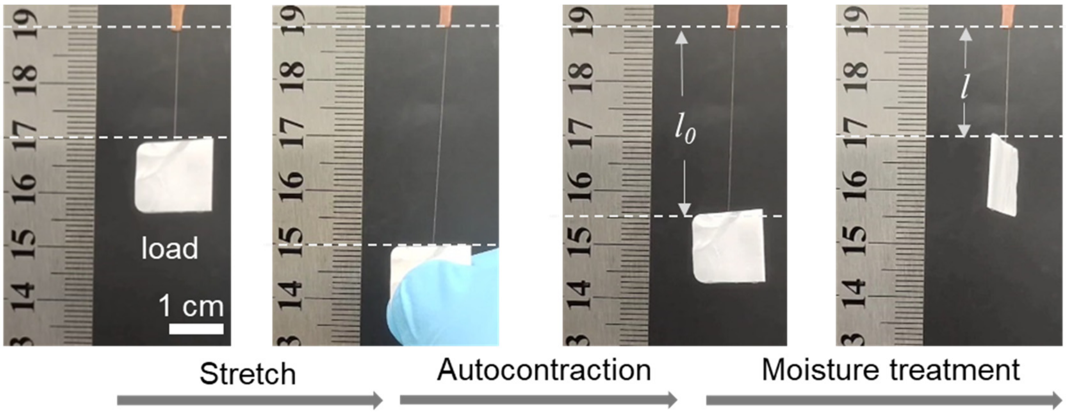

Supplementary Fig. 30. Photos for measuring load-dependent supercontraction ratios. Typically, a 2-cm-length hydrogel microfiber tied to a specific load was stretched to 4 cm (100% strain), and then after removing the restraint, the fiber autocontracted to a length of l_0. Moisture treatment further shortened the fiber to l. The supercontraction ratio was defined as $(l_0 - l)/l_0$.

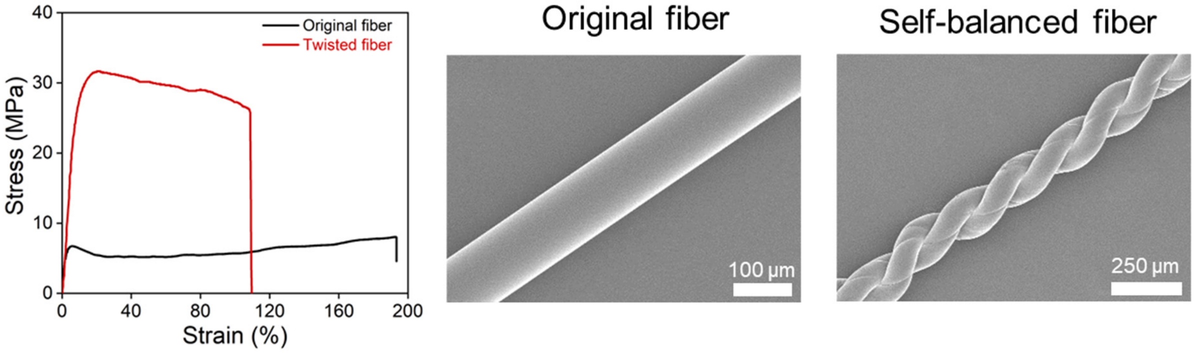

Supplementary Fig. 31. Tensile stress-strain curves and corresponding SEM images of original and twisted hydrogel microfibers.

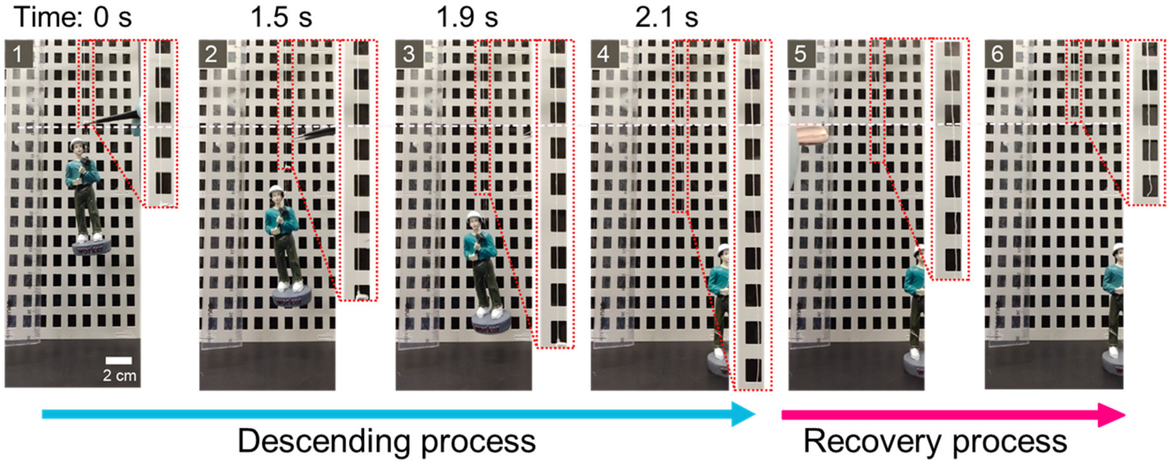

Supplementary Fig. 32. Photos of the descending and moisture-induced recovery processes of the twisted hydrogel microfiber as the rescue rope.

Supplementary Table 1. Comparison of the combinatory properties of spider silk and PDMAEA-Q/PMAA hydrogel microfiber. Note that, the data for PDMAEA-Q/PMAA hydrogel microfiber were recorded at ambient conditions (25 ºC, RH 60%). The moisture sensitivity and fracture energy of spider silk were calculated in this work (Supplementary Figs. S20, 23).

Properties	Spider silk	PDMAEA-Q/PMAA hydrogel microfiber
**Elongation**	15-45%[S3, 4]	219%
**Stiffness/modulus**	3-10 GPa[S5]	428 MPa
**Water content**	~9.3 wt%[S3]	~31.4 wt%
**Moisture sensitivity**	~0.19 wt%/RH%	~0.5 wt%/RH%
**Crack-resistant**	Yes[S6]	Yes
**Fracture energy**	735 kJ m^{-2}	187 kJ m^{-2}
**Damping capacity**	50-70%[S7]	95%
**Self-healability**	Partly[S8]	96%, 30 s
**Supercontraction**	50%[S9]	43%
**Spinning method**	Pultrusion spinning	Pultrusion spinning
**Solvent**	Water	Water
**Scalable fabrication**	Yes	Yes
**Nanoconfinement**	β-sheet nanocrystal	H-bond cluster

Supplementary Table 2. Signs of the main cross-peaks in 2DCOS synchronous and asynchronous spectra.

Cross-peak signs (left: syn; right: asyn)				
1615	+, -	-, +	+, +	
1630	+, -	-, +		
1691	-, +			
1730				
	1730	1691	1630	1615

Sign multiplication (syn×asyn)				
1615	-	-	+	
1630	-	-		
1691	-			
1730				
	1730	1691	1630	1615

According to Noda's rule, the order of different wavenumbers is: 1630 cm^{-1} → 1615 cm^{-1} → 1691 cm^{-1} → 1730 cm^{-1}, i.e. δ(OH) ($H_2O\cdots$PDMAEA-Q) → δ(OH) ($H_2O\cdots$PMAA) → ν(COOH) (H-bond cluster of PMAA) → ν(C=O) (PDMAEA-Q).

Supplementary Table 3. Comparison of the fracture energies and elongations among PDMAEA-Q/PMAA hydrogel microfiber and other materials.

Materials		Fracture energy (kJ m^{-2})	Elongation (%)	Ref.
**PDMAEA-Q/PMAA hydrogel microfiber**		187	219	This work
**Spider silk**		735	39	
**Human tissues**	Skin	1.7-2.6	54±17	[S10]
	Cartilage	0.14-1.16	5.4±1.6	[S11]
	Bone	0.25-0.57	1.9±0.6	[S12]
**Wood**	Thuya wood	0.5-3.7	5.3	[S13]
**Plastics**	CF/PEI	3.17-4.07	60	[S14]
	PEI	4.5±0.9	78±0.7	[S15]
	EPDM/PP TPE	10.4±0.9	300	[S16]
**Elastomers**	Hybrid network elastomer	13.5	170	[S17]
	PDM elastomers	12	1800	[S18]
**Composite materials**	Glass fabric composite hydrogel 1	250	50	[S19]
	Glass fabric composite hydrogel 2	1000	10	[S20]
	Metallic glass composite	340	13	[S21]
	Carbon/glass fibre composite 1	137-288	2.3	[S22]
	Carbon/glass fibre composite 2	118-360	4.5±0.5	[S23]
**Metal alloys**	Copper alloy	3-241	40-50	[S24]
	Aluminum alloy	22	6.3-9.8	[S25]
**Hydrogels**	PAAm/alginate hydrogel	9	2300	[S26]
	Polyampholyte hydrogel	4	700	[S27]
	PDMA-g-PNIPAM hydrogel	0.8	950	[S28]
	PNIPAM-co-FOSA hydrogel	8	1300	[S29]
**Ionogels**	Microsphere-reinforced ionogel	87	3250	[S30]
	P(AAm-co-AA) ionogel	24	600	[S31]
**Spider silk-inspired materials**	PVA/HCPE composite	130	550	[S32]
	IPDI-SPU elastomer	215	1520	[S33]
	Octuple HB elastomer	17	360	[S34]

Supplementary Table 4. Comparison of the properties and preparing conditions among typical artificial thin fibers. N/A denotes "not available" in the literature.

Fibers	Elongation (%)	Tough-ness (MJ/m^3)	Damping capacity (%)	Supercontraction (%)	Self-heal	Fracture energy (kJ/m^2)	Diameter (μm)	Solvent	Spinning method	Ref.
**PDMAEA-Q/PMAA**	219	19.8	95	43	96% 30 s	187	30-250	H_2O	Pultrusion or draw spinning	This work
**PCL-b-PEG-b-PCL/P5-Azo-P5**	N/A	N/A	N/A	50	N/A	N/A	3-20	$CHCl_3$		[S35]
**Host-guest hydrogel**	24	~16	75	50	No	N/A	6.5	H_2O		[S36]
	18	~23	72	N/A	N/A	N/A	3.1-12.8	H_2O		[S37]
**PHEAAm**	30	~20	N/A	N/A	N/A	N/A	~30	Ethanol/ H_2O		[S38]
**HA network**	50	~2.7	N/A	75	N/A	N/A	4-20	H_2O		[S39]
**Silica/PAA hydrogel**	44	370	95	90	No	N/A	10-500	H_2O		[S40]
	55	383	95	75	No	N/A	5-50	H_2O		[S41]
**Core–shell MAPAH**	1200	26.8	N/A	No	No	N/A	20-300	DMSO /H_2O		[S42]
**P(AAm-co-AA)/Fe**	500	~3	22	No	No	N/A	30-120	Glycerol / H_2O		[S43]
**Lignin hydrogel**	86.5	281.6	N/A	N/A	N/A	N/A	15-50	H_2O		[S44]
**Protein organogel**	527	371	N/A	N/A	Yes	N/A	~30	THF		[S45]
**Polymorphic regenerated silk**	20	~15	N/A	N/A	N/A	N/A	5-10	HFIP		[S46]
**Pseudoprotein**	750	387	N/A	N/A	N/A	N/A	30	HFIP		[S47]
**PEO or nylon 66**	450	~550	N/A	N/A	N/A	N/A	0.2-1.6	CH_3CN		[S48]
**Polyglycerol fiber**	~3.5	~4.5	N/A	N/A	N/A	N/A	~30	H_2O		[S49]
**Recombinant protein**	~25	130	N/A	N/A	No	N/A	30	H_2O	Wet spinning	[S50]
**GO/MWNT/PU**	673	543.7	N/A	60	No	N/A	~25	Ethanol/ H_2O		[S51]
**Polyaniline**	4	~22	N/A	No	No	N/A	5	Cresol/ DMF		[S52]
**PS-b-PEO**	900	121.2	N/A	70	N/A	N/A	68-192	THF/ H_2O		[S53]
**PANa fiber**	1090	4.8	N/A	N/A	N/A	N/A	550-1100	H_2O/ methanol		[S54]
**Alginate/HEA organohydrogel**	405	~0.4	N/A	N/A	No	N/A	1000	Glycerol /H_2O		[S55]
**PNA hydrogel**	900%	~10.2	N/A	N/A	Yes	N/A	560-1521	EtOAc		[S56]
**Bacterial cellulose**	295	116.3	N/A	N/A	N/A	N/A	500	H_2O	Wet drawing	[S57]
**Ca-alginate**	N/A	N/A	N/A	N/A	No	N/A	100-220	H_2O	Microfluidic spinning	[S58]
**P/n-type TE**	15	~3	N/A	N/A	N/A	N/A	1600	H_2O		[S59]
**PAMPS/PAAm**	159	~8.5	N/A	N/A	N/A	N/A	590-2430	H_2O	Reactive spinning	[S60]
**PDMS/PAAm**	520	~5	N/A	N/A	No	N/A	~1000	H_2O	Tube templating	[S61]
**PAAm/PDMS/ZnS:Cu**	250	~0.09	N/A	N/A	No	N/A	~4000	H_2O		[S62]
**Ionic organogel**	190	~1.5	N/A	No	No	N/A	>600	Ethylene glycol		[S63]

Supplementary references

S1. Noda, I. Two-dimensional infrared (2D IR) spectroscopy: Theory and applications. *Appl. Spectrosc.* **44**, 550-561 (1990).

S2. Sun, S. & Wu, P. Spectral insights into microdynamics of thermoresponsive polymers from the perspective of two-dimensional correlation spectroscopy. *Chin. J. Polym. Sci.* **35**, 700-712 (2017).

S3. Yazawa, K., Malay, A. D., Masunaga, H., Norma-Rashid, Y. & Numata, K. Simultaneous effect of strain rate and humidity on the structure and mechanical behavior of spider silk. *Commun. Mater.* **1**, 10 (2020).

S4. Rising, A. & Johansson, J. Toward spinning artificial spider silk. *Nat. Chem. Biol.* **11**, 309-315 (2015).

S5. Yarger, J. L., Cherry, B. R. & van der Vaart, A. Uncovering the structure–function relationship in spider silk. *Nat. Rev. Mater.* **3**, 18008 (2018).

S6. Giesa, T., Arslan, M., Pugno, N. M. & Buehler, M. J. Nanoconfinement of spider silk fibrils begets superior strength, extensibility, and toughness. *Nano Lett.* **11**, 5038-5046 (2011).

S7. Kelly, S. P., Sensenig, A., Lorentz, K. A. & Blackledge, T. A. Damping capacity is evolutionarily conserved in the radial silk of orb-weaving spiders. *Zool.* **114**, 233-238 (2011).

S8. Keten, S., Xu, Z., Ihle, B. & Buehler, M. J. Nanoconfinement controls stiffness, strength and mechanical toughness of β-sheet crystals in silk. *Nat. Mater.* **9**, 359-367 (2010).

S9. Blackledge, T. A. *et al.* How super is supercontraction? Persistent versus cyclic responses to humidity in spider dragline silk. *J. Exp. Biol.* **212**, 1981-1989 (2009).

S10. Pereira, B. P., Lucas, P. W. & SweeHin, T. Ranking the fracture toughness of thin mammalian soft tissues using the scissors cutting test. *J. Biomech.* **30**, 91-94 (1997).

S11. ChinPurcell, M. V. & Lewis, J. L. Fracture of articular cartilage. *J. Biomech. Eng.* **118**, 545-556 (1996).

S12. Pope, M. H. & Murphy, M. C. Fracture energy of bone in a shear mode. *Med. Biol. Eng.* **12**, 763-767 (1974).

S13. Alami, S. E. *et al.* Fracture energy of wood and root burl wood of Thuya (Tetraclinis articulata). *J. Trop. For. Sci.* **25**, 166-174 (2013).

S14. Kim, K., Ye, L. & Yan, C. Fracture behavior of polyetherimide (PEI) and interlaminar fracture of CF/PEI laminates at elevated temperatures. *Polym. Composite.* **26**, 20-28

(2005).
S15. In Moon, S., Monson, L. L. & Extrand, C. W. Insert-molded poly(ether imide)/carbon fiber poly(ether ether ketone) bimaterial composites: Fracture and interfacial analysis. *J. Appl. Polym. Sci.* **102**, 2362-2371 (2006).
S16. Wang, C. & Chang, C. I. Fracture energies and tensile strength of an EPDM/PP thermoplastic elastomer. *J. Appl. Polym. Sci.* **75**, 1033-1044 (2000).
S17. Wu, J., Cai, L. & Weitz, D. A. Tough self-healing elastomers by molecular enforced integration of covalent and reversible networks. *Adv. Mater.* **29**, 1702616 (2017).
S18. Kang, J. *et al.* Tough and water-insensitive self-healing elastomer for robust electronic skin. *Adv. Mater.* **30**, 1706846 (2018).
S19. King, D. R. *et al.* Extremely tough composites from fabric reinforced polyampholyte hydrogels. *Mater. Horiz.* **2**, 584-591 (2015).
S20. Huang, Y. *et al.* Superior fracture resistance of fiber reinforced polyampholyte hydrogels achieved by extraordinarily large energy-dissipative process zones. *J. Mater. Chem. A*. **7**, 13431-13440 (2019).
S21. Hofmann, D. C. *et al.* Designing metallic glass matrix composites with high toughness and tensile ductility. *Nature* **451**, 1085-1089 (2008).
S22. Kirk, J. N., Munro, M. & Beaumont, P. W. R. The fracture energy of hybrid carbon and glass fibre composites. *J. Mater. Sci.* **13**, 2197-2204 (1978).
S23. Munro, M. & Lai, C. P. Z. The elevated-temperature dependence of fracture energy mechanisms of hybrid carbon-glass fibre reinforced composites. *J. Mater. Sci.* **23**, 3129-3136 (1988).
S24. Alexander, D. J., Zinkle, S. J. & Rowcliffe, A. F. Fracture toughness of copper-base alloys for fusion energy applications. *J. Nucl. Mater.* **271**, 429-434 (1999).
S25. Johnson, F. A., Glover, A. P. & Radon, J. C. Fracture toughness and fracture energy measurements on aluminium alloys. *Eng. Fract. Mech.* **8**, 381-390 (1976).
S26. Sun, J. *et al.* Highly stretchable and tough hydrogels. *Nature* **489**, 133-136 (2012).
S27. Sun, T. L. *et al.* Physical hydrogels composed of polyampholytes demonstrate high toughness and viscoelasticity. *Nat. Mater.* **12**, 932-937 (2013).
S28. Hu, Y. *et al.* Hydrophilicity-hydrophobicity transformation, thermoresponsive morphomechanics, and crack multifurcation revealed by AIEgens in mechanically strong hydrogels. *Adv. Mater.* **33**, 2101500 (2021).
S29. Wang, F. & Weiss, R. A. Thermoresponsive supramolecular hydrogels with high fracture toughness. *Macromolecules* **51**, 7386-7395 (2018).

S30. Li, W. *et al.* Recyclable, healable, and tough ionogels insensitive to crack propagation. *Adv. Mater.* **34**, 2203049 (2022).

S31. Wang, M. *et al.* Tough and stretchable ionogels by in situ phase separation. *Nat. Mater.* **21**, 359-365 (2022).

S32. Liu, L. *et al.* Dynamic nanoconfinement enabled highly stretchable and supratough polymeric materials with desirable healability and biocompatibility. *Adv. Mater.* **33**, 2105829 (2021).

S33. Li, Z. *et al.* Healable and recyclable elastomers with record-high mechanical robustness, unprecedented crack tolerance, and superhigh elastic restorability. *Adv. Mater.* **33**, 2101498 (2021).

S34. Zhuo, Y. *et al.* Simultaneously toughening and stiffening elastomers with octuple hydrogen bonding. *Adv. Mater.* **33**, 2008523 (2021).

S35. Li, J., Wang, H., Liu, B., Chen, J., Gu, J. & Lin, S. Photoinduced contraction fibers and photoswitchable adhesives generated by stretchable supramolecular gel. *Adv. Funct. Mater.* **34**, 2201851 (2022).

S36. Wu, Y. *et al.* Biomimetic supramolecular fibers exhibit water-induced supercontraction. *Adv. Mater.* **30**, 1707169 (2018).

S37. Wu, Y. *et al.* Bioinspired supramolecular fibers drawn from a multiphase self-assembled hydrogel. *Proc. Natl. Acad. Sci. U.S.A.* **114**, 8163-8168 (2017).

S38. Cruz, M. V., Shah, D. U., Warner, N. C., McCune, J. A. & Scherman, O. A. Facile, energy-efficient microscale fibrillation of polyacrylamides under ambient conditions. *Adv. Mater.* **34**, 2201577 (2022).

S39. Chu, C. K. *et al.* Chemical tuning of fibers drawn from extensible hyaluronic acid networks. *J. Am. Chem. Soc.* **142**, 19715-19721 (2020).

S40. Dou, Y. *et al.* Artificial spider silk from ion-doped and twisted core-sheath hydrogel fibres. *Nat. Commun.* **10**, 5293 (2019).

S41. He, W. *et al.* Protein-like nanogel for spinning hierarchically structured artificial spider silk. *Adv. Mater.* **34**, 2201843 (2022).

S42. Zhao, X., Chen, F., Li, Y., Lu, H., Zhang, N. & Ma, M. Bioinspired ultra-stretchable and anti-freezing conductive hydrogel fibers with ordered and reversible polymer chain alignment. *Nat. Commun.* **9**, 3579 (2018).

S43. Ju, M., Wu, B., Sun, S. & Wu, P. Redox-active iron-citrate complex regulated robust coating-free hydrogel microfiber net with high environmental tolerance and sensitivity. *Adv. Funct. Mater.* **30**, 1910387 (2020).

S44. He, X. *et al.* Ultrastretchable, adhesive, and antibacterial hydrogel with robust spinnability for manufacturing strong hydrogel micro/nanofibers. *Small* **17**, 2103521 (2021).

S45. Ma, C. *et al.* Anisotropic protein organofibers encoded with extraordinary mechanical behavior for cellular mechanobiology applications. *Angew. Chem. Int. Ed.* **59**, 21481-21487 (2020).

S46. Ling, S., Qin, Z., Li, C., Huang, W., Kaplan, D. L. & Buehler, M. J. Polymorphic regenerated silk fibers assembled through bioinspired spinning. *Nat. Commun.* **8**, 1387 (2017).

S47. Gu, L., Jiang, Y. & Hu, J. Scalable spider-silk-like supertough fibers using a pseudoprotein polymer. *Adv. Mater.* **31**, 1904311 (2019).

S48. Liao, S. *et al.* Draw-spinning of kilometer-long and highly stretchable polymer submicrometer fibers. *Adv. Sci.* **4**, 1600480 (2017).

S49. Yang, H.-S. *et al.* Preparation of self-healable and spinnable hydrogel by dynamic boronate ester bond from hyperbranched polyglycerol and boronic acid-containing polymer. *Macromol. Res.* **29**, 140-148 (2021).

S50. Li, Y. *et al.* Bioinspired and mechanically strong fibers based on engineered non-spider chimeric proteins. *Angew. Chem. Int. Ed.* **59**, 8148-8152 (2020).

S51. Kim, H. *et al.* Bio-inspired stretchable and contractible tough fiber by the hybridization of GO/MWNT/polyurethane. *ACS Appl. Mater. Interfaces*. **11**, 31162-31168 (2019).

S52. Fang, B. *et al.* Scalable production of ultrafine polyaniline fibres for tactile organic electrochemical transistors. *Nat. Commun.* **13**, 2101 (2022).

S53. Lang, C. *et al.* Nanostructured block copolymer muscles. *Nat. Nanotechnol.* **17**, 752-758 (2022).

S54. Wang, X.-Q. *et al.* Macromolecule conformational shaping for extreme mechanical programming of polymorphic hydrogel fibers. *Nat. Commun.* **13**, 3369 (2022).

S55. Song, J. *et al.* Mechanically and electronically robust transparent organohydrogel fibers. *Adv. Mater.* **32**, 1906994 (2020).

S56. Shuai, L., Guo, Z. H., Zhang, P., Wan, J., Pu, X. & Wang, Z. L. Stretchable, self-healing, conductive hydrogel fibers for strain sensing and triboelectric energy-harvesting smart textiles. *Nano Energy* **78**, 105389 (2020).

S57. Guan, Q.-F. *et al.* Bio-inspired lotus-fiber-like spiral hydrogel bacterial cellulose fibers. *Nano Lett.* **21**, 952-958 (2021).

S58. Kang, E., Jeong, G. S., Choi, Y. Y., Lee, K. H., Khademhosseini, A. & Lee, S.-H. Digitally tunable physicochemical coding of material composition and topography in continuous microfibres. *Nat. Mater.* **10**, 877-883 (2011).

S59. Ding, T. *et al.* Scalable thermoelectric fibers for multifunctional textile-electronics. *Nat. Commun.* **11**, 6006 (2020).

S60. Duan, X. *et al.* Large-scale spinning approach to engineering knittable hydrogel fiber for soft robots. *ACS Nano* **14**, 14929-14938 (2020).

S61. Wang, C., Wu, B., Sun, S. & Wu, P. Interface deformable, thermally sensitive hydrogel–elastomer hybrid fiber for versatile underwater sensing. *Adv. Mater. Technol.* **5**, 2000515 (2020).

S62. Yang, C., Cheng, S., Yao, X., Nian, G., Liu, Q. & Suo, Z. Ionotronic luminescent fibers, fabrics, and other configurations. *Adv. Mater.* **32**, 2005545 (2020).

S63. Lee, Y. *et al.* Ionic spiderwebs. *Sci. Rob.* **5**, eaaz5405 (2020).
