# Supplementary material for: Aqueous spinning of robust, self-healable, and crack-resistant hydrogel microfibers enabled by hydrogen bond nanoconfinement
Source: Nat Commun. 2023 Mar 13;14:1370. doi: 10.1038/s41467-023-37036-4 (PMC10011413; doi:10.1038/s41467-023-37036-4)
Supplement: Supplementary file 2 — Description of Additional Supplementary Files [file 41467_2023_37036_MOESM2_ESM.pdf]

Description of Additional Supplementary Files

File Name: Supplementary Movie 1
Description: Aqueous pultrusion spinning of PDMAEA-Q/PMAA hydrogel microfiber

File Name: Supplementary Movie 2
Description: . Load capacity of PDMAEA-Q/PMAA hydrogel microfiber

File Name: Supplementary Movie 3
Description: Moisture-induced self-healing of PDMAEA-Q/PMAA hydrogel microfiber

File Name: Supplementary Movie 4
Description: Measurement of load-dependent supercontraction ratios

File Name: Supplementary Movie 5
Description: Moisture-induced supercontraction of hydrogel microfiber web
